# Structure of trimeric pre-fusion rabies virus glycoprotein in complex with two protective antibodies

**DOI:** 10.1016/j.chom.2022.07.014

**Published:** 2022-09-14

**Authors:** Weng M. Ng, Sofiya Fedosyuk, Solomon English, Gilles Augusto, Adam Berg, Luke Thorley, Anna-Sophie Haselon, Rameswara R. Segireddy, Thomas A. Bowden, Alexander D. Douglas

**Affiliations:** 1 Jenner Institute, Old Road Campus Research Building, Roosevelt Drive, Oxford OX3 7DQ, UK; 2 Division of Structural Biology, Wellcome Centre for Human Genetics, University of Oxford, Roosevelt Drive, Oxford OX3 7BN, UK

**Keywords:** rabies virus, glycoprotein, structure, antibody neutralization, viral fusion

## Abstract

Rabies virus (RABV) causes lethal encephalitis and is responsible for approximately 60,000 deaths per year. As the sole virion-surface protein, the rabies virus glycoprotein (RABV-G) mediates host-cell entry. RABV-G’s pre-fusion trimeric conformation displays epitopes bound by protective neutralizing antibodies that can be induced by vaccination or passively administered for post-exposure prophylaxis. We report a 2.8-Å structure of a RABV-G trimer in the pre-fusion conformation, in complex with two neutralizing and protective monoclonal antibodies, 17C7 and 1112-1, that recognize distinct epitopes. One of these antibodies is a licensed prophylactic (17C7, Rabishield), which we show locks the protein in pre-fusion conformation. Targeted mutations can similarly stabilize RABV-G in the pre-fusion conformation, a key step toward structure-guided vaccine design. These data reveal the higher-order architecture of a key therapeutic target and the structural basis of neutralization by antibodies binding two key antigenic sites, and this will facilitate the development of improved vaccines and prophylactic antibodies.

## Highlights

• 2.8-Å cryo-EM structure of trimeric pre-fusion rabies virus glycoprotein
• Structure reveals trimerization interface and binding of two neutralizing antibodies
• Conformational transition can be inhibited by both antibodies and targeted mutation

## Graphical abstract

**Figure:**
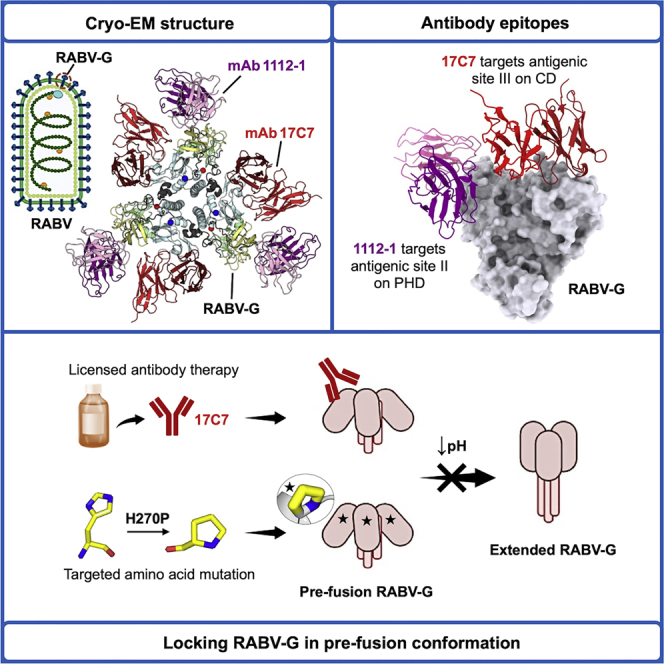

## Teaser

Ng, Fedosyuk et al. present the structure of a complex of pre-fusion trimeric rabies glycoprotein (RABV-G) with two neutralizing antibodies, one of which (17C7) is used clinically for post-exposure prophylaxis. They show that 17C7 inhibits pre- to post-fusion conformational transition, and they identify amino acid substitutions with a similar effect.

## Introduction

An estimated 3 billion people live at risk of rabies virus (RABV; genus *Lyssavirus* and family *Rhabdoviridae*) infection, which causes fatal encephalitis (Hampson et al., 2015; Fooks et al., 2017). Although effective pre- and post-exposure vaccines and passive immunization treatments are available, their high cost and need for multiple doses to achieve protection result in inadequate coverage of at-risk populations (World Health Organization, 2018).

The RABV envelope surface displays the virion glycoprotein RABV glycoprotein (RABV-G), a trimeric class III viral fusion protein, which mediates receptor binding and membrane fusion during host-cell entry. Multiple host proteins have been implicated in RABV cell entry in different contexts, but no single RABV-G:receptor interaction has been shown to be indispensable across contexts (Lentz et al., 1982; Tuffereau et al., 2007; Thoulouze et al., 1998; Wang et al., 2018b). In contrast to class I viral fusion proteins, which are also trimeric and are better studied, RABV-G can transition reversibly between a pre-fusion form (predominant at neutral pH) and post-fusion form (predominant at acidic pH) (Gaudin et al., 1991, 1992).

As the sole virion-surface protein, RABV-G is the primary target of protective antibodies (Gaudin et al., 1999; Wiktor et al., 1973). Historically, polyclonal rabies immune globulin (RIG) has been used for post-exposure passive immunization. The use of anti-RABV-G neutralizing monoclonal antibodies (mAbs) is now attracting increasing attention as an alternative to expensive and often human donor-derived RIG (Sparrow et al., 2019). Two anti-rabies mAb products have been licensed for clinical use in India, which experiences more rabies cases than any other single country: one is a single antibody (Rabishield, 17C7 or RAB1; Sloan et al., 2007; Gogtay et al., 2018); the other is a combination of two mAbs (Twinrab, docaravimab/miromavimab, also known as 62-71-3 and M777; Müller et al., 2009). Draft guidance to industry regarding the path to potential approval of such mAbs for the US market has recently been published by the US FDA (FDA, 2021). A key consideration in the development of such therapies is the breadth of coverage against circulating rabies isolates and ideally also against related bat lyssaviruses, which also cause human disease. In this regard, there is some concern about the vulnerability of 17C7, administered as a single mAb, to known antigenic polymorphisms (De Benedictis et al., 2016; Sloan et al., 2007).

Development of subunit protein and mRNA-based rabies vaccines has, so far, proven challenging (Aldrich et al., 2021; Ertl, 2019). It is believed that the trimeric pre-fusion form is the ideal immunogen, as its surface displays the major known neutralizing antibody (nAb) epitopes and monomeric proteins have performed poorly as immunogens (De Benedictis et al., 2016; Koraka et al., 2014). To our knowledge, however, production of soluble and stable pre-fusion trimeric recombinant RABV-G has not been reported. Previous efforts to structurally characterize RABV-G produced crystal structures of a single domain and monomeric ectodomains of RABV-G in complex with nAbs, verifying that RABV-G is a class III fusion glycoprotein (Hellert et al., 2020; Yang et al., 2020). Extensive efforts over decades have, however, been unsuccessful in obtaining high resolution insight into either the protein’s higher-order architecture or complexes of nAbs with the biologically and antigenically critical pre-fusion conformation. This information would empower the rational design of improved RABV vaccine immunogens and antibody-based therapeutic cocktails.

## Results

### Structure of trimeric pre-fusion RABV-G

To characterize trimeric RABV-G, we recombinantly expressed a construct of the full-length G trimer (Lys1-Leu505) encoding a site-directed mutation (H270P), which we designed with the intention of stabilizing the pre-fusion conformation. His270 lies within an elongated alpha-helix in a previous crystal structure of monomeric RABV-G ectodomain (RABV-G^ecto^) at low pH (likely post-fusion conformation), and a pre-fusion stabilizing effect of the analogous L271P mutation upon the glycoprotein of another rhabdovirus (vesicular stomatitis virus [VSV]) has previously been reported (Yang et al., 2020; Ferlin et al., 2014).

**Figure 1:**
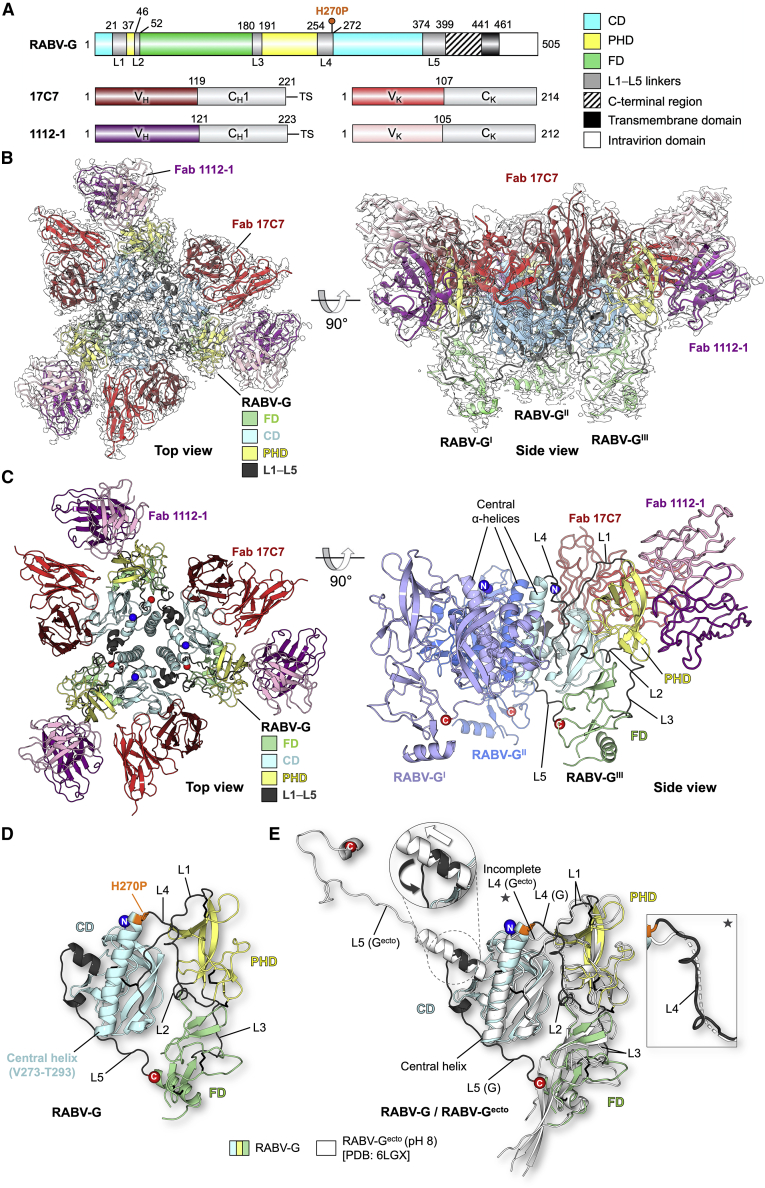
Structure of pre-fusion RABV-G trimer in complex with Fabs 17C7 and 1112-1 (A) Schematic of RABV-G domain boundaries. Linear map of RABV-G protein sequence is drawn to scale using DOG software (Ren et al., 2009), with domains colored as indicated in the legend, showing “palindromic” architecture typical of class III fusion proteins (CD, central domain; PHD, pleckstrin homology domain; FD, fusion domain). The H270P point mutation in our protein construct is indicated with a pin above the map. C-terminal region (shaded) is unresolved in our structure. (Bottom) Linear maps of Fabs 17C7 and 1112-1 heavy and kappa light chains, colored and labeled accordingly. V_H_, V_K_, C_H1_, and C_K_ denote the antibody variable heavy, variable kappa light, constant 1 heavy, and constant kappa light-chain domains, respectively. TS, TwinStrep tag. (B) 2.8-Å cryo-EM map with the resulting structure of trimeric RABV-G shown in top and side view orientations. Single copies of 17C7 and 1112-1 were observed to bind to each protomer of RABV-G. The atomic model is fitted into the corresponding cryo-EM map (white) and colored according to domain with the variable regions of Fab 17C7 and Fab 1112-1 colored red and purple (darker shade for heavy chain, lighter shade for light chain), respectively. The constant regions of the Fabs were disordered in the reconstruction and therefore were not built. (C) Atomic model of the RABV-G-Fab 17C7-Fab 1112-1 complex. (Top view) The protein molecules are displayed in cartoon representation and colored accordingly as labeled. N- and C-termini are shown as blue and red spheres, respectively. (Side view) Only one copy of each Fab is shown in ribbon representation. Two RABV-G protomers are colored blue and light purple for visual clarity. The remaining copy is colored as shown in top view. (D) Conformational features revealed by atomic model of RABV-G. A single protomer of RABV-G is shown in cartoon representation. PHD is colored yellow, CD in blue, and FD in green, whereas the inter-domain linkers (L1−L5) are colored in dark gray. N- and C-termini are shown as blue and red spheres, respectively. The point mutation H270P is colored orange and shown in stick representation. (E) Structure superimposition of our trimeric RABV-G with a previously reported monomeric RABV-G ectodomain structure obtained at pH 8.0. (RABV-G^ecto^, white cartoon, PDB: 6LGX) (Yang et al., 2020). When domains were aligned separately, the PHD and CD aligned more closely than the FD (calculated root-mean-square deviations 1.0-Å over 72 equiv C⍺ atoms for PHD, and 0.6-Å/128 C⍺ for CD, and 2.4-Å/88 C⍺ for FD). Differences in the L4 and L5 linkers between our trimeric RABV-G structure and the previous RABV-G^ecto^ structure are highlighted in the inset. For further information, see Figures S1–S3 and Tables S1 and S2.

Detergent-extracted RABV-G was complexed with the antigen-binding fragments (Fab) of mAb 17C7 (described above) and mAb 1112-1. mAb 1112-1 is a well-characterized antibody that has been a leading contender for inclusion in mAb-based post-exposure prophylaxis cocktail and binds an epitope distinct from that of 17C7 (Dietzschold et al., 1992; Müller et al., 2009). Characteristics of all antibodies used in this study are summarized in Table S1. The complex was subjected to single-particle cryo-electron microscopy (cryo-EM) analysis to generate a 2.8-Å resolution structure of a RABV-G trimer-Fab 17C7-Fab 1112-1 complex (Figure 1; Figures S1 and S2; Table S2).

As observed for other class III fusogens (Roche et al., 2006), each protomer of the trimer is composed of three domains: a membrane-distal pleckstrin homology domain (PHD), a membrane-proximal fusion domain (FD), and a laterally positioned central domain (CD) (Figures 1D and 1E). Topologically, PHD sits atop the FD, and the CD is located adjacent to the PHD/FD junction. The PHD is connected to the CD via two linkers Leu22-Asn37 (termed L1) and Gly255-Leu271 (L4), respectively, and to the FD via two separate linkers consisting of residues Lys47-Ser52 (L2) and Glu181-Ile191 (L3). The fusion loops, transmembrane, and intraviral regions were not resolved in the reconstruction, indicative that these regions of the molecule exhibit elevated levels of flexibility in the purified protein.

Structural overlay of our trimeric RABV-G with the previously reported monomeric RABV-G^ecto^ determined at pH 8.0 reveals good structural agreement between individual domains, but significant dissimilarity in inter-domain linkers, rearrangement of which is believed to be critical for the pre- to post-fusion conformational transition (Figure 1E; Roche et al., 2007). The region from Pro374 to the C terminus in the high-pH crystal structure of G^ecto^ forms a helical structure distal from the FD. We term the equivalent region of our trimeric RABV-G “L5,” extending from Pro374 to the C-terminal limit of our model at Leu399. L5 forms a loop which interacts with the adjacent protomer’s CD and then runs back across the base of the CD to reach the FD (Figure S3). The interaction interface between the L5 loop and the FD contains several hydrophobic interactions, including contributions by a cluster of histidines (His86, His173, and His397; Figure S3A).

A second notable difference between trimeric RABV-G and the RABV-G^ecto^ pH 8.0 crystal structure lies in linker L4, which connects the central helix of the CD with the PHD. Although the linker was not fully observed in the G^ecto^ structure, it is resolved in our structure due to the stabilizing contacts mediated by the trimeric organization of the molecule (Figure 1E; Figure S3C). This region forms an ⍺-helix in the previously reported low-pH RABV-G^ecto^ structure and contains both the helix-breaking H270P substitution introduced here and a series of further amino acids, which have previously been implicated in the fusion competence of the protein (His261, Asp266, and Glu269) (Yang et al., 2020). Conservation of the local architecture in the region of Pro270 in our structure, compared with the pre-fusion VSV-G structure and high-pH RABV-G^ecto^ crystal structure (Figure 1E; Figure S4; Roche et al., 2007; Yang et al., 2020), suggests that this region of our protein retains an authentic conformation. We observe that L4 mediates a series of hydrophobic contacts with the PHD and FD and hydrogen bonds with L1 Asn26 and the central helix (Figure S3D).

**Figure 2:**
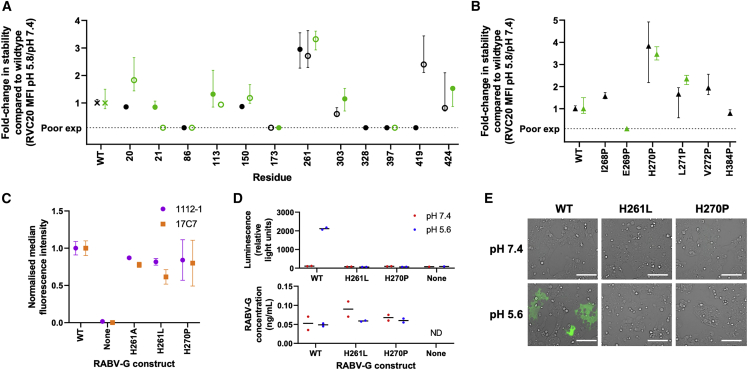
Targeted mutations at two sites in L4 stabilize pre-fusion RABV-G Wild-type (WT) and mutant RABV-G constructs were expressed on transiently transfected Expi293 cells, and reactivity with site I (RVC20), site II (1112-1), and site III (17C7) IgG was assessed by flow cytometry. Cell-surface expression levels of all constructs are shown in Table S3. (A) Effect of histidine substitutions. All histidine residues in the RABV-G ectodomain were mutated to alanine and to leucine, with exceptions detailed in methods. Pre-fusion protein stability was measured by calculating median fluorescence intensity (MFI) of the pre-fusion-specific mAb RVC20 (Hellert et al., 2020; De Benedictis et al., 2016) after binding at pH 5.8 as a proportion of that after binding to the same construct at pH 7.4: this proportion was 0.11 for untagged WT RABV-G, and 0.12 for WT RABV-G expressed as a fusion protein. Results are expressed as fold change in this proportion compared with WT protein. Filled and open symbols denote introduction of alanine and leucine, respectively. Black and green symbols denote untagged constructs and those expressed as GFP fusion proteins, respectively. Points represent median and error bars represent range of four technical replicates across two experiments (a transfection with each of two independent DNA preparations on each of 2 days). “Poor exp” denotes constructs with cell-surface expression <33% of the level of WT RABV-G, as assessed by RVC20 binding at pH 7.4 (Table S3). (B) Effect of potentially helix-breaking substitutions with proline. Residues in L4/L5 regions expected to form helices in post-fusion protein were substituted with proline. Colors, replication (points and error bars), and the definition of poor cell-surface expression are as for (A). (C) H261A/L and H270P retain site II and site III antigenicity, as evidenced by 1112-1 and 17C7 binding. Untagged constructs were used. MFI is expressed as a proportion of that observed with WT RABV-G with each antibody. “None” denotes MFI on cells transfected with an irrelevant antigen. The replication strategy and meaning of points and error bars were as for (A). (D and E) H261L and H270P mutations abolish RABV-G-mediated cell-cell fusion. Acid-triggered cell-cell fusion was monitored in a dual-reporter luminescence and fluorescence assay. (D) shows luciferase activity (upper graph) and expression level measured by ELISA (lower graph) for samples from the same experiment. Points each represent median of three or more technical replicates (as described in STAR Methods section) using a single DNA preparation. Line indicates median of biological replicates using independent DNA preparations. ND indicates not detectable. (E) shows GFP activity, imaged by fluorescence microscopy. Scale bars, 100 μm.

To further explore the role of L4 and L5 in pH-mediated conformational transition, we used a flow cytometry-based assay to assess the effect of targeted mutations on conformational stability of RABV-G (Figure 2). Of 12 targeted histidine loci across the protein, H261A/L mutations (in L4) had the greatest stabilizing effect (a 3-fold increase in retention of the pre-fusion conformation at pH 5.8). These mutations would prevent the electrostatic interaction observed between His261 and Asp211 in the low-pH G^ecto^ structure, and their effect suggests that the His261-Asn26 hydrogen bond is dispensable for the pre-fusion structure. Attempted mutations at the L5-FD interface proved incompatible with cell-surface protein expression (Table S3).

We also assessed the effect of substitution with proline of each residue from Ile268 to Val272 (in L4) and of His384 (in L5). On the basis of comparison to previous low-pH RABV-G^ecto^, Chandipura virus G (CHAV-G), and VSV-G structures (Yang et al., 2020; Baquero et al., 2015; Roche et al., 2006), these residues are likely to lie in extended helices in the post-fusion conformation, formation of which may be disrupted by proline. H270P substitution had a marked stabilizing effect (3- to 4-fold increase in retention of pre-fusion conformation at pH 5.8) (Figure 2B).

To test the effect of conformationally stabilizing mutations upon RABV-G’s function, we performed a cell-cell fusion assay. Both H270P and H261L abolished RABV-G’s fusion competence (Figures 2D and 2E). Loss of function of H261A in a similar assay has also been reported (Yang et al., 2020).

H270P and H261L both preserved approximately wild-type (WT) cell-surface expression levels and antigenicity (Figure 2C; Table S3) and represent important steps toward structure-guided design of stable RABV-G subunit vaccines. The information we provide regarding L4 and L5 structure should facilitate design of improved future constructs.

### Trimerization of RABV-G

**Figure 3:**
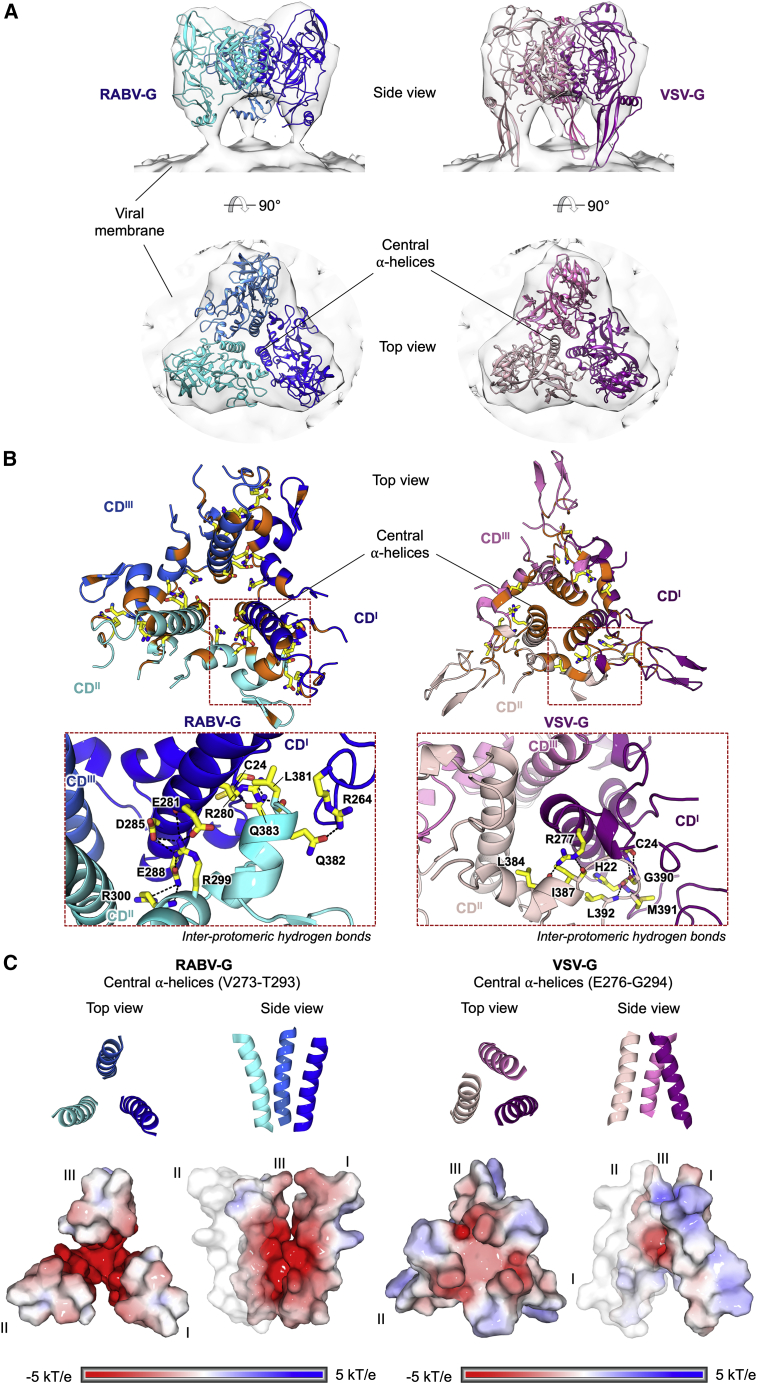
Structural comparison of pre-fusion RABV-G and VSV-G reveals contrasting modes of inter-protomeric interactions at the trimerization core (A) Fitting of pre-fusion RABV-G (blue) and VSV-G (pink; PDB: 5I2S) into a subtomographic average map of pre-fusion VSV-G (semi-transparent gray; EMD-9331) (Si et al., 2018). RABV-G and VSV-G structures are shown in cartoon representation and colored in different shades of blue and pink, respectively. Maps are shown as transparent surfaces. (B) Zoom-in views of the G trimerization core involved in inter-protomeric interactions. Residues involved in hydrogen bonding (black dashed lines) are shown as sticks, with the carbon, nitrogen, oxygen, and sulfur constituents colored yellow, blue, red, and dark yellow, respectively. Residues involved in non-polar interactions are colored orange. The zoom-in panels of the CD α-helices shows the inter-protomeric hydrogen bonds. This analysis demonstrates that the trimerization core of RABV-G is largely mediated by polar interactions between charged residues, including a network of hydrogen bonds formed by negatively charged Glu281, Asp285, and Glu288 on one protomer and positively charged Arg299 and Arg300 on the adjacent protomer. Hydrophobic interactions are formed at the periphery and bottom of the central α-helices. In contrast, VSV-G displays the reverse pattern of inter-protomeric interactions, where the core is largely maintained by hydrophobic interactions with hydrogen bonds formed at the periphery. (C) Electrostatic potential of the central α-helices. The central α-helices of RABV-G (left) and VSV-G (right) are shown as cartoons (top) and surfaces (bottom). The surfaces are colored according to the electrostatic potential in the range of ±5 kT/e, as calculated by adaptive Poisson-Boltzmann solver (APBS). Both RABV-G and VSV-G display negatively charged trimerization cores. This characteristic is especially prominent in RABV-G, where the carboxyl groups of Glu274, Glu275, Glu281, Glu282, Asp285, and Glu288 side chains line the central α-helices.

In our structure, RABV-G monomers assemble as a compact tripod with the CDs located adjacent to the trimerization axis (Figure 3). Each CD presents a near vertically oriented ⍺-helix (Val273-Thr293) that packs and forms the trimerization core. Similar to VSV-G, the extent of the inter-protomeric interface (∼1,600 Å^2^) is approximately 1/7 of that in a pre-fusion herpesvirus class III fusion protein, human cytomegalovirus gB (∼11,200 Å^2^) (Liu et al., 2021).

Other features of the RABV-G trimerization interface, however, contrast with the pre-fusion VSV-G structure. Notably, the central helices in our RABV-G structure form an inverted cone with an electronegative core and the apex proximal to the membrane, whereas in VSV-G, the apex of the analogous cone is distal to the membrane (Figure 3C). This reflects differing relative angulation of the entire protomers between the two structures (i.e., the orientation of the RABV-G central helix relative to the remainder of the RABV-G protomer in our structure is similar to that seen in VSV-G) (Figure 3A; Figure S4). In addition to the contributions of the L4 and L5 loops to intra-protomeric interactions (Figure S3), as described above, the inter-protomeric interactions include hydrogen bonds formed by Leu381, Gln382, and Gln383 (within L5) with Arg280 (within central helix), Arg264 (within L4), and Cys24 (within L1), respectively (Figure 3B).

### Structural basis of RABV-G binding by protective antibodies

Previous antibody epitope mapping studies have revealed three major antigenic sites on RABV-G, designated I−III, and two less-commonly recognized sites, IV and “a” (Benmansour et al., 1991; Kuzmina et al., 2013; Figure S5). Here, we reveal the molecular specificity underlying antibody-mediated targeting by the Fabs of two mAbs, 1112-1 and 17C7, which are each protective against RABV challenge in animal models and bind to sites II and III, respectively (Sloan et al., 2007; Dietzschold et al., 1992).

**Figure 4:**
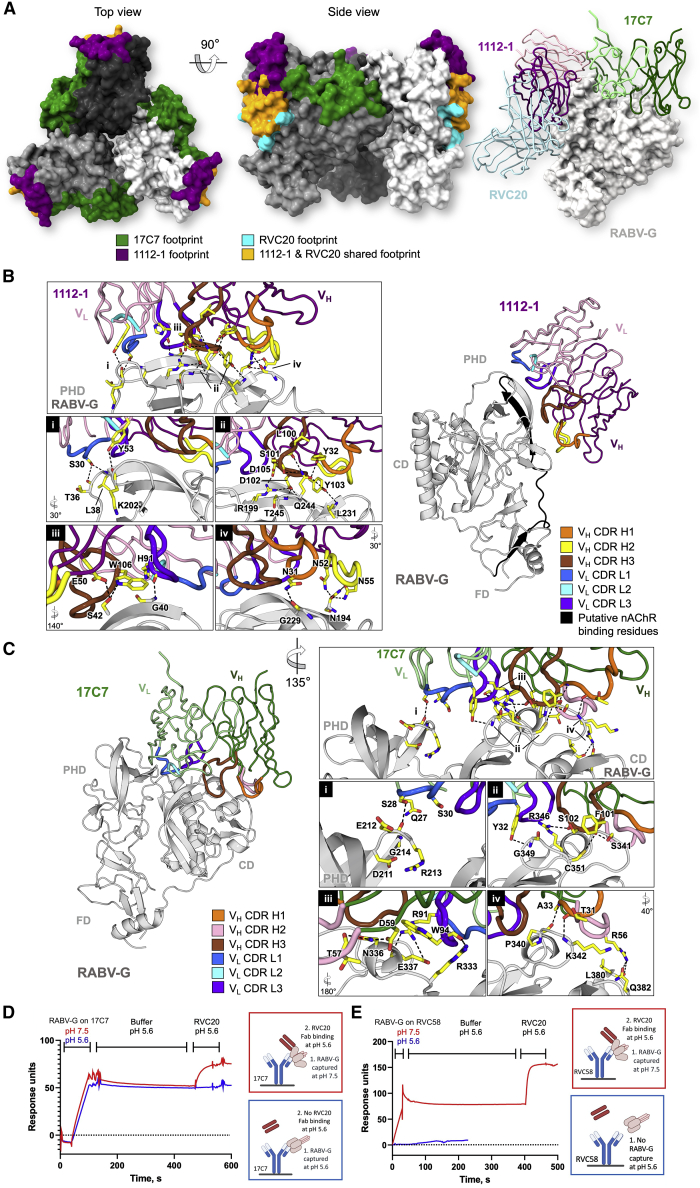
Structural basis for antibody-mediated RABV neutralization: multiple sites of vulnerability on the membrane-distal crown of the RABV-G trimer (A) Visualization of structurally characterized anti-RABV antibody epitopes. (Left) Footprints exhibited by RVC20 (site I-targeting antibody, PDB: 6TOU; cyan), 1112-1 (site II; purple), and 17C7 (site III; green) plotted on trimeric RABV-G (white, gray, and dark gray surface). MAbs 1112-1 and RVC20 are shown to target distinct yet overlapping epitopes (orange surface). For clarity, the variable regions of the Fab fragments of mAbs RVC20, 1112-1, and 17C7 bound to a single RABV-G protomer are shown as ribbons (right). MAbs (B) 1112-1 and (C) 17C7 target the PHD and CD domains of RABV-G, respectively. Detailed interactions between complementarity-determining regions (CDRs) of each antibody with RABV-G (gray cartoon) are highlighted in the boxed panels. Residues involved in the antibody-antigen interactions are shown as yellow sticks, with the CDR loops colored as indicated. In our structure, one molecule of each Fab binds to each protomer of the RABV-G trimer. (B) The 1112-1 epitope encompasses three β strands and five loops on the PHD, which include residues 175-203 (highlighted black) that have been previously implicated in nicotinic acetylcholine receptor (nAChR) recognition by RABV (Lentz, 1990). RABV-G residues Asn194, Arg199, Gln244, and Thr245 form extensive hydrogen bond networks with 1112-1 CDR residues including Asn52, Asn55, Ser101, Asp102, Tyr103, and Asp105. Detailed interactions are provided in Figure S8. (C) 17C7 has been observed to bind mostly to the CD in addition to a small contact with the PHD. Notably, 17C7 engages residues Asn336 and Arg346. Mutations at these locations have been shown to confer resistance to 17C7 neutralization (Sloan et al., 2007; Wang et al., 2011). The 17C7 light chain also forms hydrogen bonds with Lys330 and Arg333, which have been reported to play roles in recognition of the receptor p75NTR (Tuffereau et al., 1998) and in neuroinvasion by RABV (Coulon et al., 1998). Detailed interactions are provided in Figure S8. (D and E) Locking of RABV-G in pre-fusion conformation by 17C7 and RVC58. SPR traces demonstrating that when RABV-G is captured by site-III-binding mAbs 17C7 or RVC58, respectively, at pH 7.4, followed by incubation at pH 5.6, binding of the pre-fusion conformation-specific RVC20 Fab remains possible. RVC20 and RVC58 are specific for the pre-fusion conformation: RVC58 fails to capture RABV-G at pH 5.6, and RVC20 does not bind at pH 5.6, unless RABV-G had previously been captured by 17C7 in pre-fusion conformation. Figure S9E demonstrates similar results obtained with a different assay format, in which the initial interaction between the site-III-binding antibody and RABV-G occurs in solution, and the conformation-specific RVC20 Fab is immobilized on the chip surface. See Figures S5–S9 and Table S1 for more related information.

In our structure, Fabs 17C7 and 1112-1 bind to each protomer of the RABV-G trimer (Figure 4). Fab 17C7 binds CD with an interaction interface nearly parallel to the membrane, whereas Fab 1112-1 engages the PHD with an interface nearly perpendicular to the membrane. This alternating longitudinal and latitudinal binding to adjacent epitopes brings the two Fabs in close proximity, such that contacts are observed between the 17C7 light chain and the 1112-1 heavy and light chains (Figure S5C). No ordered density corresponding to glycans was seen in our map (Figure S6), but the 1112-1 epitope is adjacent to the potential glycosylation sites Asn37 (believed to have low glycan occupancy; Yamada et al., 2013) and Asn247.

The mAb 1112-1 epitope encompasses three β strands and five loops in the PHD (Figure 4B), described in detail in Figures S7 and S8. The epitope includes but also extends well beyond the classically described site II (residues 34−42 and 198−200) (Kuzmina et al., 2013) and overlaps with that of mAb RVC20 (as visualized in a previous crystal structure of RVC20 in complex with the isolated RABV-G PHD) (Figure 4A; Figure S5; Hellert et al., 2020). We confirmed by competition enzyme-linked immunosorbent assay (ELISA) that 1112-1 and RVC20 cannot bind RABV-G simultaneously (Figure S5D).

The mAb 17C7 epitope encompasses both the CD and, to a lesser extent, the PHD (Figure 4C; Figures S7 and S8). It includes Asn336 and Arg346 in RABV-G, mutation of which have been shown to confer relative resistance to 17C7 neutralization in the CVS strain of the virus (Sloan et al., 2007; Wang et al., 2011): our structure reveals that these residues form hydrogen bonds with 17C7 paratope residues. The epitope also includes several additional residues that vary between phylogroup I lyssavirus species residues (Figure S7). The mAb 17C7 light chain also forms hydrogen bonds with Lys330 and Arg333, which have been reported to be important for recognition of the neurotrophin receptor, p75NTR, and in neuroinvasion by RABV (Tuffereau et al., 1998; Coulon et al., 1998).

Given the clinical importance of 17C7, we sought to explore its mechanism of action. Due to the presence of Arg333 within site III and the known importance of this residue in neurovirulence and p75NTR binding, it was previously postulated that site-III-binding antibodies may function by blocking RABV-G binding to p75NTR (Yang et al., 2020; Dietzschold et al., 1983). However, p75NTR is dispensable for RABV-G pathogenicity (Tuffereau et al., 2007), suggesting this is unlikely to be the only mechanism of action of such antibodies. In contrast, the site I-binding mAb RVC20 has previously been suggested to act in a receptor-independent manner, by “locking” RABV-G in pre-fusion conformation. Although RVC20 bound (post-fusion) RABV-G poorly at low pH, the rate of RVC20 dissociation from RABV-G was pH-independent if association had occurred at neutral pH (Hellert et al., 2020).

Mapping the 17C7 epitope onto the low-pH-derived G^ecto^ structure (Yang et al., 2020) revealed that although the contact residues are likely to remain largely accessible in the post-fusion conformation, the acidic-pH-induced structural transition may separate the major (CD) and minor (PHD) regions of the bipartite epitope by more than 20 Å (Figure S5B). We hypothesized that binding of 17C7 may hinder this separation and hence prevent the pre- to post-fusion conformational transition in RABV-G.

We confirmed the conformational specificity of RVC20, 1112-1, and 17C7 (sites I, II, and III mAbs, respectively), using a surface plasmon resonance (SPR) assay (Figure S9). Consistent with our and other data (Hellert et al., 2020), RVC20 binding was abrogated at low pH. Despite appreciable reduction in their binding affinity compared with that at pH 7.4, 1112-1 and 17C7 remained capable of binding RABV-G with nanomolar affinity at pH 5.6 (Figure S9D). In the case of 17C7, this is consistent with the fact that both CD and PHD elements of the binding site remain accessible on the RABV-G^ecto^ structure, and PHD makes a relatively minor contribution to the binding footprint.

As 17C7 binding in itself could not be used as a reporter of RABV-G conformation, we used a “sandwich-configuration” SPR assay to assess the effect of 17C7 upon pH-induced RABV-G conformational change (Figure 4D). We also assessed the effect of another site-III binding mAb, RVC58 (De Benedictis et al., 2016), which unlike 17C7 appears not to bind post-fusion RABV-G (Figure 4E). When RABV-G had been captured by either 17C7 or RVC58 under neutral conditions, followed by lowering of the pH to 5.6 for 300 s, RVC20 remained able to bind despite being applied at pH 5.6. We observed similar results when antibody binding to RABV-G took place in solution, rather than on the chip surface (Figure S9E). The pH-triggered conformational change which abrogates RVC20 binding is thus inhibited when RABV-G has already been bound by either site III mAb (17C7 or RVC58) in pH-neutral/pre-fusion conformation.

## Discussion

Given the health and economic burden of rabies disease and recent progression in vaccine technology, there is both need and opportunity to develop more immunogenic and more cost-effective rabies vaccines. Design of highly expressed and stable pre-fusion RABV-G trimers would considerably assist production of such vaccines.

Together with another report in close agreement with our data, our data reveals the structure of a pre-fusion lyssavirus glycoprotein trimer (Figure 1). It resolves the RABV-G trimerization interface, L4 linker, and likely authentic pre-fusion conformation of L5 (Figure 3; Figure S3), each of which plays an important role in the maintenance of the pre-fusion architecture and transition to post-fusion conformation and complements another recent report (Callaway et al., 2022).

Transient dissociation of the RABV-G trimer is believed to be required to permit rearrangement to the post-fusion conformation (Albertini et al., 2012). A dynamic equilibrium between trimeric and monomeric forms is known to exist for VSV-G at neutral pH, presumably facilitating this rearrangement (Zagouras et al., 1991; Lyles et al., 1990). The electronegative inner surfaces of the helix bundles, and the lack of stabilizing interactions between those bundles (Figure 3C), suggest relatively weak inter-protomeric interactions, compatible with a similar equilibrium for RABV-G.

L4 is one of the few regions of RABV-G known to form new secondary structure during the transition to the post-fusion conformation (Yang et al., 2020). Our data shows that in the pre-fusion conformation, L4 mediates contacts between the CD and the PHD (Figure S3). Separation of these contacts would be necessary to allow hinging movement of the PHD relative to the CD, which is thought to be required during rearrangement of class III proteins into post-fusion conformation (Roche et al., 2007). This may be facilitated by protonation of His261, disrupting the pre-fusion hydrogen bond with Asn26 in L1, and allowing formation of the interaction with Asp211 as previously visualized in RABV-G^ecto^ low-pH structure (Yang et al., 2020).

Similarly, the interface between L5 and the FD observed in our structure is likely to separate during pre-to-post-fusion conformational transition. The cluster of histidine residues seen at this interface (His86, His173, and His397; Figure S3A) may be protonated at low pH and hence act as a “pH sensor,” disrupting the L5-FD interface and initiating the movement of FD toward the target membrane. RABV-G His397 corresponds to VSV-G His407, which lies within a similar cluster of histidine residues and is known to function as a pH sensitive switch during VSV-G conformational transition (Beilstein et al., 2020). The different orientations of the L5 region in our RABV-G and RABV-G^ecto^ are similar to the pre-fusion and intermediate conformations of VSV-G and CHAV-G, respectively (Roche et al., 2007; Baquero et al., 2017). It seems possible that the RABV-G^ecto^ structure, which lacks membrane interactions and is potentially influenced by the packing environment of the protein crystal, may represent such an intermediate. The conformations of L4 and L5 revealed by our structure thus give clues about the roles these regions play during the fusogenic conformational change of RABV-G, where dislocation of the L5 loop from the FD and separation of the L4 from the PHD are required to create an extended intermediate. This is consistent with a model based upon low-pH structures of RABV-G and other rhabdovirus glycoproteins, whereby L4 residues from 266 onward then extend the central helix, with an adjacent antiparallel helix formed by re-folding of L5 (Yang et al., 2020; Albertini et al., 2012; Baquero et al., 2015; Belot et al., 2020).

Given the important roles of L4, L5, and the trimerization interface, each is likely to be a target of structure-guided immunogen design (Graham et al., 2019). Stabilization of protein in a desired conformation is clearly critical for recombinant protein-based vaccines, especially where these require soluble forms of proteins which normally exist in the context of a viral envelope (McLellan et al., 2013). Our findings that H261A/L and H270P substitutions increase pre-fusion conformational stability are important initial steps toward this goal. Achieving soluble expression of RABV-G is likely to require further mutations, perhaps targeting the trimerization and L5-FD interfaces to favor the trimeric architecture over the monomeric form reported as RABV-G^ecto^. For mRNA or virus-vectored vaccines, which allow the expression of protein in the context of a membrane, the need for conformational stabilization has been more contentious, especially in the high-profile case of SARS-CoV-2 spike (Bos et al., 2020; Watanabe et al., 2021). To improve upon the WT RABV-G transgenes used by leading vectored rabies vaccine candidates (Aldrich et al., 2021; Wang et al., 2018a) (Jenkin et al., 2022), increased levels of antigen expression may be as important as modulation of the conformation displayed.

We also present structures of protective antibodies bound to two of RABV-G’s three major antigenic sites. Notably, these data make clear the structural basis of the known vulnerability of the licensed therapeutic, 17C7, to polymorphisms between RABV isolates and between RABV and other lyssaviruses (including bat lyssaviruses within the relatively closely related phylogroup I, which are known to cause human disease) (De Benedictis et al., 2016). This should facilitate the development of more broadly neutralizing mAbs and highlight the known risk that antigenic variation poses to efficacy of single-mAb-based post-exposure prophylaxis: the World Health Organization and others have previously encouraged development of products containing two or more mAbs with non-overlapping epitopes (Sparrow et al., 2019).

Finally, we show that two site-III-binding nAbs (17C7 and RVC58) are capable of locking RABV-G in the pre-fusion conformation. In contrast with the previous suggestion that site-III-binding antibodies may act by blockade of RABV-G-p75NTR interaction, which is dispensable for virulence (Yang et al., 2020; Tuffereau et al., 2007), antibody-mediated blockade of the conformational transition is likely to be effective against entry to all cell types. This builds upon similar previous findings with the site-I-binding antibody RVC20.

Our data do not exclude the possibility that mAbs capable of blocking conformational transition may additionally block receptor binding. It remains the case that despite at least four proteins having been postulated to act as RABV-G receptors (Tuffereau et al., 1998; Thoulouze et al., 1998; Lentz et al., 1982; Wang et al., 2018b), there is little or no structural information regarding any RABV-G:receptor complex. Broader understanding of the mechanism of action of this important therapeutic class—and in particular, resolution of the contributions of blockade of receptor binding and conformational transition—would be assisted by further structural and functional data regarding RABV-G’s interactions with the multiple proteins which may act as host receptors. Our data and the previous findings regarding RVC20 do however raise the intriguing question of whether blockade of conformational transition may be a mechanism widely shared among RABV-G-binding nAbs and independent of (but not necessarily exclusive of) any effect upon receptor interaction.

Together, our findings will facilitate rational development of improved vaccine immunogens and therapeutics against this major human and animal pathogen and extend understanding of the mechanism by which protective antibodies can neutralize the virus.

## STAR★Methods

### Key resources table

**Table:** 

REAGENT or RESOURCE	SOURCE	IDENTIFIER
**Antibodies**		

Alkaline phosphatase conjugated goat anti-mouse IgG (Fc specific)	Sigma	A1418

**Chemicals, peptides, and recombinant proteins**		

n-octyl-β-d-glucoside	Generon	O311S
A8-35 amphipol	Anatrace	A835
RABV-G wt protein C-tagged	This paper	Based on Addgene #74288
RABV-G H270P mutant protein	This paper	Based on Addgene #74288
1112-1 IgG and Fab	This paper	N/A
RVC20 IgG and Fab	This paper	N/A
17C7 IgG and Fab	This paper	N/A
RVC58 Fab	This paper	N/A
Mouse polyclonal serum from mice immunized with Rabipur rabies vaccine	This paper	N/A

**Critical commercial assays**		

Pierce Mouse IgG1 Fab and F(ab')2 Preparation kit	ThermoFisher Scientific	44980
ExpiFectamine™ 293 Transfection Kit	ThermoFisher Scientific	A14524
Alexa Fluor 647 conjugation kit	ThermoFisher Scientific	A20186
EnduRen substrate	Promega	E6481
Horseradish-peroxidase-conjugated Streptactin	Iba Life Sciences	2-1502-001

**Deposited data**		

Structure of RABV-G in complex with 17C7 and 1112-1 Fabs	PDB ID: 8A1E	https://www.wwpdb.org/
Map of RABV-G in complex with 17C7 and 1112-1 Fabs	EMDB ID: EMD-15073	https://www.emdataresource.org/

**Experimental models: Cell lines**		

Expi293F cells	ThermoFisher Scientific	A14527
GripTite™ 293 MSR	ThermoFisher Scientific	R79507
1112-1 hybridoma	Wistar Institute, USA	N/A
RABV-G H270P-mutant protein stable cell line	This paper based on Elegheert et al. (2018)	N/A

**Oligonucleotides**		

Primers for RABV-G sequence amplification from Addgene plasmid #74288: F: TAGTAGGCGGCCGCCATG GTCCCACAGGCTCTCC R: TAGTAGTCTAGATTTACGCTTC CGGTTCGAGCCGTGTCTCGCCCCC	This paper	N/A

**Recombinant DNA**		

pVIP-ENTR	Balazs et al., 2013	N/A
pOPINVH	Nettleship et al., 2008	Addgene #26041
pOPINVL	Nettleship et al., 2008	Addgene #26040
pHR-CMV-TetO2-3C-Twin-Strep-IRES-Turquoise2	Elegheert et al., 2018	Addgene #113886
Plasmids for heavy and light chains of 1112-1 Fab	Synthesized	N/A
Plasmids for heavy and light chains of RVC20 Fab	Synthesized based on patent	WO2016078761
Plasmids for heavy and light chains of RVC20 IgG	Synthesized based on patent	WO2016078761
Plasmids for heavy and light chains of 17C7 Fab	Synthesized based on patent	WO2006084006
Plasmids for heavy and light chains of 17C7 IgG	Synthesized based on patent	WO2006084006
Plasmids for heavy and light chains of RVC58 Fab	Synthesized based on patent	WO2016078761
pDSP1-7	Synthesized based on Kondo et al. (2010)	N/A
pDSP8-11	Synthesized based on Kondo et al. (2010)	N/A
Plasmid for RABV-G wt protein C-tagged	Modified with C-tag, based on sequence from Kim et al. (2016)	Based on Addgene #74288
Plasmid for RABV-G wt protein	Synthesized based on sequence from Kim et al. (2016)	Based on Addgene #74288
Plasmid for RABV-G-GFP wt protein	Synthesized based on sequence from Kim et al. (2016)	Based on Addgene #74288
Plasmid for RABV-G-GFP H20L mutant protein	Synthesized based on sequence from Kim et al. (2016)	Based on Addgene #74288
Plasmid for RABV-G H20A mutant protein	Synthesized based on sequence from Kim et al. (2016)	Based on Addgene #74288
Plasmid for RABV-G-GFP H21L mutant protein	Synthesized based on sequence from Kim et al. (2016)	Based on Addgene #74288
Plasmid for RABV-G-GFP H21A mutant protein	Synthesized based on sequence from Kim et al. (2016)	Based on Addgene #74288
Plasmid for RABV-G-GFP H86L mutant protein	Synthesized based on sequence from Kim et al. (2016)	Based on Addgene #74288
Plasmid for RABV-G H86A mutant protein	Synthesized based on sequence from Kim et al. (2016)	Based on Addgene #74288
Plasmid for RABV-G-GFP H113L mutant protein	Synthesized based on sequence from Kim et al. (2016)	Based on Addgene #74288
Plasmid for RABV-G-GFP H113A mutant protein	Synthesized based on sequence from Kim et al. (2016)	Based on Addgene #74288
Plasmid for RABV-G H150A mutant protein	Synthesized based on sequence from Kim et al. (2016)	Based on Addgene #74288
Plasmid for RABV-G-GFP H150L mutant protein	Synthesized based on sequence from Kim et al. (2016)	Based on Addgene #74288
Plasmid for RABV-G H173L mutant protein	Synthesized based on sequence from Kim et al. (2016)	Based on Addgene #74288
Plasmid for RABV-G-GFP H173A mutant protein	Synthesized based on sequence from Kim et al. (2016)	Based on Addgene #74288
Plasmid for RABV-G H261L mutant protein	Synthesized based on sequence from Kim et al. (2016)	Based on Addgene #74288
Plasmid for RABV-G H261A mutant protein	Synthesized based on sequence from Kim et al. (2016)	Based on Addgene #74288
Plasmid for RABV-G-GFP H261L mutant protein	Synthesized based on sequence from Kim et al. (2016)	Based on Addgene #74288
Plasmid for RABV-G H303L mutant protein	Synthesized based on sequence from Kim et al. (2016)	Based on Addgene #74288
Plasmid for RABV-G-GFP H303A mutant protein	Synthesized based on sequence from Kim et al. (2016)	Based on Addgene #74288
Plasmid for RABV-G H328A mutant protein	Synthesized based on sequence from Kim et al. (2016)	Based on Addgene #74288
Plasmid for RABV-G H397A mutant protein	Synthesized based on sequence from Kim et al. (2016)	Based on Addgene #74288
Plasmid for RABV-G-GFP H397L mutant protein	Synthesized based on sequence from Kim et al. (2016)	Based on Addgene #74288
Plasmid for RABV-G H419A mutant protein	Synthesized based on sequence from Kim et al. (2016)	Based on Addgene #74288
Plasmid for RABV-G H419L mutant protein	Synthesized based on sequence from Kim et al. (2016)	Based on Addgene #74288
Plasmid for RABV-G H424L mutant protein	Synthesized based on sequence from Kim et al. (2016)	Based on Addgene #74288
Plasmid for RABV-G-GFP H424A mutant protein	Synthesized based on sequence from Kim et al. (2016)	Based on Addgene #74288
Plasmid for RABV-G I268P mutant protein	Synthesized based on sequence from Kim et al. (2016)	Based on Addgene #74288
Plasmid for RABV-G-GFP E269P mutant protein	Synthesized based on sequence from Kim et al. (2016)	Based on Addgene #74288
Plasmid for RABV-G H270P mutant protein	Synthesized based on sequence from Kim et al. (2016)	Based on Addgene #74288
Plasmid for RABV-G-GFP H270P mutant protein	Synthesized based on sequence from Kim et al. (2016)	Based on Addgene #74288
Plasmid for RABV-G L271P mutant protein	Synthesized based on sequence from Kim et al. (2016)	Based on Addgene #74288
Plasmid for RABV-G-GFP L271P mutant protein	Synthesized based on sequence from Kim et al. (2016)	Based on Addgene #74288
Plasmid for RABV-G L272P mutant protein	Synthesized based on sequence from Kim et al. (2016)	Based on Addgene #74288
Plasmid for RABV-G H384P mutant protein	Synthesized based on sequence from Kim et al. (2016)	Based on Addgene #74288

**Software and algorithms**		

ImageJ	(Schneider et al., 2012)	https://imagej.nih.gov/ij/
cryoSPARC	Punjani et al. (2017)	https://cryosparc.com/
COOT v.0.8.9.2	Emsley and Cowtan (2004)	https://www2.mrc-lmb.cam.ac.uk/personal/pemsley/coot/
Phenix v.1.19.2	Adams et al. (2002)	https://phenix-online.org/
MolProbity v.4.5.1	Chen et al. (2010)	http://molprobity.biochem.duke.edu/
FlowJo v10 software	BD Biosciences	https://www.flowjo.com/
Biacore S200 v2 Evaluation software	Cytiva	N/A
Prism 9.0	GraphPad Software	https://www.graphpad.com/
PyMOL	Schrodinger	pymol.org
UCSF Chimera/Chimera X	Pettersen et al., 2004; Pettersen et al., 2021	https://www.cgl.ucsf.edu/chimera/; https://www.rbvi.ucsf.edu/chimerax/
BioRender	BioRender	https://biorender.com/

**Other**		

96-well white tissue culture plates	PerkinElmer	6005680
μ-Plate black 96-well plates	Ibidi	89626
CM5 chip	Cytiva	29104988
Biacore S200 instrument	Cytiva	N/A
Strep-Tactin®XT 4Flow® high capacity cartridge	Iba Life Sciences	2-5027-001

### Resource availability

#### Lead contact

Further information and requests for resources and reagents should be directed to and will be fulfilled by the lead contact, Alexander D Douglas (sandy.douglas@ndm.ox.ac.uk).

#### Materials availability

All novel materials produced in this paper materials are available on request from the lead contact, subject to a mutually acceptable materials transfer agreement.

### Experimental model and subject details

#### Cells

The Expi293F suspension cell line (ThermoFisher Scientific, cat. #A14527) was grown at 37°C in 5% CO_2_ in Expi293 Expression Medium (ThermoFisher Scientific) at 130 rpm agitation.

The GripTite™ 293 MSR cells (ThermoFisher Scientific, cat. #R79507) were grown at 37°C in 5% CO_2_ in Dulbecco’s Modified Eagle’s Medium-high glucose (Sigma-Aldrich, D6546), supplemented with 10% heat-inactivated fetal bovine serum (FBS), 100 U/mL penicillin, 100 μg/ml streptomycin, 4mM L-glutamine, 0.1 mM MEM Non-Essential Amino Acids and 600 μg/mL of Geneticin. For transfection, all antibiotics were removed from the above mentioned medium.

RABV-G H270P-mutant protein stable cell line was grown at 37°C in 5% CO_2_ in BalanCD293 medium (Fujifilm-Irvine Scientific) supplemented with 4 mM GlutaMAX, 1% Pen-Strep solution and with or without 5% BalanCD feed (Fujifilm-Irvine Scientific) at 130 rpm agitation.

1112-1 hybridoma (gift from Prof. Hildegund Ertl, Wistar Institute, USA), was cultured at 37°C in 5% CO_2_ using a CELLine CL 1000 Bioreactor (Cole-Parmer) in Dulbecco’s Modified Eagle’s Medium-high glucose (Sigma-Aldrich, D6546) supplemented with 100 U/mL penicillin, 100 μg/ml streptomycin, and 4mM L-glutamine. For cell compartment, medium was supplemented with 20% of ultra-low IgG fetal bovine serum.

### Method details

#### Production of antibodies and Fabs

Antibodies used in the study and their main characteristics are summarized in Table S1.

1112-1 IgG was purified from 1112-1 hybridoma supernatant on a 1 mL Protein G column (ThermoFisher Scientific) as per the manufacturer’s instructions. To prepare 1112-1 Fab for cryo-EM experiment, IgG was digested using a Mouse IgG1 Fab and F(ab')2 Preparation kit (ThermoFisher Scientific).

1112-1 heavy and light chain coding sequences were obtained by RNA sequencing (Absolute Antibody) and cloned into pVIP-ENTR, a mammalian expression vector modified from the pENTR4 vector (ThermoFisher Scientific) by incorporation of a synthetic ‘CASI’ promoter (a kind gift of Martino Bardelli) (Balazs et al., 2013). Briefly, coding sequences were synthesized (ThermoFisher Scientific) with appropriate adaptors for cloning using an In-Fusion Snap Assembly Kit (Takara Bioscience) and, for the heavy chain, C-terminal sequence encoding a TwinStrep tag (Schmidt et al., 2013). Synthetic genes were inserted into a linearized pVIP-ENTR vector using an In-Fusion Snap Assembly Kit (Takara Biosciences).

Sequences of the variable regions of RVC20, RVC58 and 17C7 have been disclosed (Thomas et al., 2010; Corti, 2016; Hellert et al., 2020). To produce the antibodies in Fab format, synthetic clonal genes carrying variable regions of the antibodies were synthesized by Twist Biosciences. Suitable adaptors were added to the genes to enable cloning into pOPINVH and pOPINVL vectors (Addgene #26041 and 26040) (Nettleship et al., 2008) using the In-Fusion Snap Assembly Kit. Heavy chain coding sequences included TwinStrep (TS) (RVC20, 17C7) or His_6_ (RVC58) purification tags. pOPINVH was linearized with AfeI and XbaI restriction enzymes for RVC20-TS and 17C7-TS heavy chain cloning, or KpnI and SfoI for RVC58-His heavy chain cloning. pOPINVL was linearized with KpnI and SacI for cloning of all light chains.

To produce plasmids encoding RVC20 and 17C7 IgG heavy chains, VH regions for RVC20 and 17C7 were PCR-amplified from the above pOPINVH-based vectors. Forward and reverse PCR primers contained suitable InFusion adapters for further InFusion cloning of the PCR fragments in frame with a human IgG1 constant region coding sequence in the pVIP-ENTR backbone.

All recombinant Fabs and IgG were expressed by transient transfection of Expi293F cells (Thermo Fisher Scientific), with a 1:1 heavy:light-chain-coding plasmid ratio, using the ExpiFectamine™ 293 Transfection Kit as per manufacturer’s recommendations. Supernatants were harvested 96 h after transfection and purified on either StrepTactin XT Superflow column (IBA Lifesciences) for RVC20-TS or 17C7-TS Fabs, Talon Superflow column (Cytiva) for RVC58-His Fab, or Protein G column (ThermoFisher Scientific) for IgGs, as per manufacturer’s recommendations.

#### Production of RABV-G H270P

To produce untagged full-length RABV-G H270P-mutant protein, a stable cell line was generated essentially as previously described (Elegheert et al., 2018), with minor modifications.

The construct was based upon one previously described, composed of a codon optimized Pasteur strain ectodomain coding sequence chimerized with a SAD-B19 strain transmembrane and intracellular domain (Kim et al., 2016) but encoding the desired H270P mutation. This was ligated into the pHR-CMV-TetO2-3C-Twin-Strep-IRES-Turquoise2 lentiviral shuttle plasmid (Addgene #113886), allowing production using a previously described method of a lentivirus encoding RABV-G H270P and mTurquoise fluorescent protein (the latter after an internal ribosome entry site), under the control of a tet-repressible promoter (Elegheert et al., 2018). HEK293-Trex cells (Thermo Fisher Scientific) which had previously been adapted for growth in suspension in CD293 medium (Thermo Fisher Scientific), were then transduced with this virus by spinoculation at 1300 x g for 2 h at 30°C. Four days after transduction, RABV-G expression was partially induced using 0.01 μg/mL tetracycline and, 24 h later, the 5% of cells with the highest level of expression of mTurquoise were collected using an SH800 cell sorter (Sony). Sorted cells were expanded, initially in static culture in progressively larger vessels from a 24-well plate, in DMEM-F12 medium (ThermoFisher Scientific), supplemented with 10% fetal bovine serum (FBS), 1% penicillin/streptomycin and 1 μg/mL blasticidin (Melford Scientific). Cells were then returned to suspension culture in Erlenmeyer flasks, initially in CD293 medium (with 10% FBS, 4 mM GlutaMAX, 1% penicillin-streptomycin solution, and 1 μg/mL blasticidin), then, over two weeks, with progressive reduction of the amount of serum to 0.5% and replacement of CD293 medium with BalanCD293 medium (Fujifilm-Irvine Scientific).

For protein expression, cells were grown to 4x10^6^ cells/mL, diluted 50/50 with fresh BalanCD293 medium supplemented with 4 mM GlutaMAX, 1% Pen-Strep solution and 5% BalanCD feed (Fujifilm-Irvine Scientific), and induced with 10 μg/mL of tetracycline for 48 h. Cell pellets were collected, resuspended in storage buffer (50 mM HEPES, pH 7.5, 150 mM NaCl and 5% glycerol), and frozen at -80°C.

To purify protein, thawed pellets were treated with extraction buffer (50 mM HEPES pH 7.5, 150 mM NaCl, 1% *n-*octyl-β-d-glucoside [Generon], cOmplete Protease Inhibitor cocktail [Roche]) supplemented with an excess of 17C7-TS Fab and Biolock solution (IBA Lifesciences) for 2 h at 4°C, and the lysate was clarified by centrifugation at 4000 x g for 30 min. RABV-G – 17C7-TS complexes were purified from clarified lysate on a Strep-Tactin XT Superflow column (IBA Lifesciences) according to the manufacturer’s recommendations, with wash and elution buffers supplemented with 1% *n-*octyl-β-d-glucoside. Fractions containing purified protein complex were mixed with 3-fold mass excess of A8-35 amphipol (Anatrace) and left at 4°C for 1 h. Detergent was then removed using Pierce detergent removal spin columns (ThermoFisher Scientific), and the resulting sample was concentrated using an Amicon centrifugal concentrator (Millipore). An excess of 1112-1 Fab, and additional 17C7-TS Fab, were then added to saturate available binding sites, and the resulting RABV-G / 17C7-TS / 1112-1 complex was purified by size-exclusion chromatography on a Superose 6 10/300 Increase column (Cytiva) in buffer containing 50 mM HEPES pH 7.5 and 150 mM NaCl.

#### Cryo-EM grid preparation

Purified RABV-G / 17C7-TS / 1112-1 complexes were concentrated and mixed with *n-*octyl-β-d-glucoside to final concentrations of 1 mg/mL and 0.07%, respectively. 4 μL of the sample was pipetted on glow-discharged Quantifoil® holey carbon grids (1.2/1.3 μm copper 200 mesh) and was blotted for 3.5 s blot time at -15 N blotting pressure prior to flash freezing in liquid ethane using a Vitrobot Mark IV (FEI/ ThermoFisher Scientific).

#### Cryo-EM data collection and processing and model building

Data acquisition was performed at the Electron Bio-Imaging Centre (eBIC) at Diamond Light Source (Harwell, UK). A 300 kV Titan Krios microscope (Thermo Fisher Scientific) equipped with a K3 direct electron detector (Gatan) and a 20 eV Gatan Imaging Filter (GIF) energy filter was used. Cryo-EM images were recorded automatically using SerialEM software in super resolution mode but saved with a 2x binning factor, at defocus values ranging from −0.8 to −2.6 μm at a calibrated magnification of 81,000x, corresponding to a pixel size of 1.06 Å/px. The total electron dose was 44.4 e^−^/Å^2^ over 45 frames (Table S2).

Recorded movies were motion corrected (global) and the contrast transfer function (CTF) parameters were determined using Gctf within cryoSPARC software (Punjani et al., 2017). Particles were automatically picked using circular blobs with a diameter of 80−150 Å. Picked particles were extracted from the micrographs with box sizes of 400 px and subsequently subjected to 2D classification. Per-particle motion correction (local) was performed on particles from selected 2D classes. *Ab-initio* reconstruction and 3D heterogeneous refinement were performed to generate 5 initial models and to classify the particles among them, respectively. Non-uniform refinement was performed on all 3D classes. Particles from the best model was used for another round of 3D heterogenous refinement (3 classes) to further classify for structural heterogeneity. The best models were selected for local (per-particle) CTF refinement to improve particle and map resolution and quality. C3 symmetry was applied for models selected for further reconstruction and refinement. Map resolution was estimated by gold-standard Fourier shell correlation (FSC) plot at the 0.143 threshold. Two maps at 2.8 Å and 3.2 Å were generated. The latter constitutes fuzzy densities around the membrane-proximal fusion domain and amphipol regions, therefore the 2.8 Å was selected for model building and refinement. The detailed data processing steps is summarized in a flowchart presented in Figure S1.

Model building for all protein chains was performed in COOT v.0.8.9.2 (Emsley and Cowtan, 2004) and refined by alternating cycles of real-space refinement in Phenix v.1.19.2 (Adams et al., 2002). Model geometry was validated using MolProbity v.4.5.1 (Chen et al., 2010). Geometry statistics, model B factors, and map vs model cross-correlation values are shown in Table S2. Map and model figures were generated using UCSF Chimera, ChimeraX (Pettersen et al., 2004, 2021), and PyMol. FSC curves and a local resolution histogram were plotted using data from cryoSPARC (Punjani et al., 2017), and correlation coefficient graphs were plotted using refinement data extracted from Phenix (Adams et al., 2002). Graphical abstract was created with figure elements exported from BioRender.com.

#### Assessment of conformational stability of RABV-G by flow cytometry

In designing potentially stabilizing substitutions, we considered both the pre-fusion structure and the likely post-fusion structure, on the basis of low pH structures of RABV-G^ecto^, VSV-G and CHAV-G (Yang et al., 2020; Roche et al., 2006; Baquero et al., 2015). Histidines have been suggested to act as pH sensors in lyssavirus glycoproteins, with protonation at low pH disrupting the pre-fusion structure. We therefore tested substitution of all histidines in the protein with alanine and leucine, with the following exceptions: for H270 and H384, substitution with proline was tested, as detailed below; for H328L, production of the expression construct failed; and H352/354 were not targeted as they do not mediate any notable interactions in the pre-fusion form and lie in regions near the protein surface not expected to be subject to rearrangement during conformational transition. We also introduced potentially helix-breaking proline substitutions at residues 266−272 and 384, which may rearrange from the L4 / L5 linkers into helices in the post-fusion protein. All evaluated substitutions are shown in Table S3.

The same RABV-G coding sequence as was used for structural studies, but with desired point mutations (and, unless otherwise specified, encoding the wildtype H270), obtained as synthetic DNA (Twist Bioscience). In some cases, constructs were synthesized in frame with sequence encoding a C-terminal flexible linker, tobacco etch virus protease cleavage site, green fluorescent protein and a 6-His tag.

The complete encoded C-terminal tag sequence in these cases was: GAGSAAGSGEFENLYFQGMVSKGEELFTGVVPILVELDGDVNGHKFSVSGEGEGDATYGKLTLKFICTTGKLPVPWPTLVTTLTYGVQCFSRYPDHMKQHDFFKSAMPEGYVQERTIFFKDDGNYKTRAEVKFEGDTLVNRIELKGIDFKEDGNILGHKLEYNYNSHNVYIMADKQKNGIKVNFKIRHNIEDGSVQLADHYQQNTPIGDGPVLLPDNHYLSTQSALSKDPNEKRDHMVLKEFVTAAGITLGMDELYKHHHHHH.

The above constructs were digested with NotI and XbaI restriction enzymes, and ligated into a similarly digested pTT3 backbone (Durocher et al., 2002).

Expi293 cells were transfected with 1 μg/mL of plasmid encoding either full-length wild-type or mutant RABV G using ExpiFectamine™ reagent (ThermoFisher Scientific) as per the manufacturer’s instructions. 300 μL of cell samples were harvested 48 h post-transfection, subjected to centrifugation at 300 x g for 5 min, and resuspended in phosphate buffered saline (PBS).

Binding of RVC20 IgG was used as an indicator of pre-fusion conformation (Figure S9; Hellert et al., 2020). 150 μL of the sample was washed in PBS (pH 7.3), whilst the other 150 μL was washed with an acidic buffer (50 mM Bis-Tris, 130 mM NaCl, pH 5.8). The samples were incubated in their respective buffer at room temperature for 30 mins. For RVC20, cell staining was performed with RVC20 IgG conjugated to Alexa Fluor 647 (Thermo Scientific), diluted in either PBS or acidic buffer to a concentration of 1 μg/mL, for 20 mins. For 1112-1 and 17C7, staining was performed similarly with IgG at 1 μg/mL, followed by secondary staining using Alexa Fluor 647-conjugated anti-mouse immunoglobulin secondary antibody (Thermo Scientific). Stained cells were subsequently washed and resuspended twice in their corresponding buffers before a final washing was repeated in PBS. Stained cells were sorted on a BD LSRFortessa™ Cell Analyzer (BD Biosciences). Using FlowJo v10 software (BD Biosciences) single cells were selected using forward scatter and side scatter-based gates, and histograms of fluorescence intensity for single cells were used to determine median fluorescence intensity (MFI) for each sample.

#### Production of wild-type RABV-G for SPR and ELISA

Surface plasmon resonance experiments used full length wildtype RABV-G with a C-terminal ‘C-tag’ (Glu-Pro-Glu-Ala). To make this, a construct was produced using a plasmid obtained from Addgene (#74288) encoding the Pasteur – SADB19 chimeric RABV-G described above (Kim et al., 2016). The insert was amplified with primers F: TAGTAGGCGGCCGCCATGGTCCCACAGGCTCTCC and R: TAGTAGTCTAGATTTACGCTTCCGGTTCGAGCCGTGTCTCGCCCCC, followed by digestion with NotI and XbaI restriction enzymes, and ligation into a similarly digested pTT3 backbone (Durocher et al., 2002).

RABV-G C-tagged wildtype protein was transiently expressed in Expi293F cells (ThermoFisher Scientific) using the ExpiFectamine™ 293 Transfection Kit as per manufacturer’s recommendations. 72 h after transfection, cell pellets were collected, resuspended in a storage buffer (50 mM HEPES, pH 7.5, 150 mM NaCl and 5% glycerol), and frozen at -80°C.

To purify protein, pellets were treated with extraction buffer (50 mM HEPES pH 7.5, 150 mM NaCl, 1% n-octyl-β-d-glucoside, and Pierce™ Protease Inhibitor Mini Tablets, EDTA-free Thermo Scientific™ (ThermoFisher Scientific)) for 2 h at 4°C, and the lysate was clarified by centrifugation at 4000 x g for 30 min. Following clarification, C-tagged RABV-G containing lysate was purified on a CaptureSelect™ C-tagXL affinity column (ThermoFisher Scientific) according to the manufacturer’s recommendations with wash and elution buffers supplemented with 1% n-octyl-β-d-glucoside. After affinity purification, elution fractions were buffer-exchanged to the extraction buffer and once again subjected to C-tag-based affinity purification. Fractions containing purified RABV-G protein were then concentrated.

With the exception of protein used in the competition ELISA shown in Figure S6D, protein was exchanged into A8-35 as described above for cryo-EM sample preparation.

With the exception of protein used for the standard curve in the ELISA shown in Figure 2D, protein was subjected to size-exclusion chromatography on a Superose 6 10/300 Increase column (Cytiva). For preparation of protein for SPR, SEC used 10 mM HEPES, 150 mM sodium chloride, 3 mM EDTA, and 0.05% polysorbate (HBS-EP+). For preparation of the protein used in the competition ELISA shown in Figure S6D, SEC used 50 mM HEPES pH 7.5, 150 mM NaCl, and 1% n-octyl-β-d-glucoside.

#### Cell-cell fusion assay

For detection of cell-cell fusion, we used the dual reporter proteins pDSP_1-7_ and pDSP_8-11_, which encode both split Renilla luciferase and split green fluorescent protein (GFP) (Kondo et al., 2010). Sequences encoding these reporters were synthesized in a vector backbone suitable for transient mammalian cell expression (pTwist CMV BetaGlobin WPRE Neo, Twist Biosciences). HEK293 cells expressing the macrophage scavenger receptor (for improved adhesion during manipulation; GripTite™ 293 MSR, Thermo Fisher Scientific) were seeded in two T25 flasks, and next day transfected with either pDSP_1-7_ or pDSP_8-11_ using Lipofectamine 2000 (Thermo Fisher Scientific) as per manufacturer’s recommendations. The following day, cells were detached, mixed and reformatted in white 96-well tissue culture plates (PerkinElmer). Cells were allowed to attach for 2 h and then transfected with 5 ng per well of plasmid encoding full-length untagged RABV-G per well (either wild type, H270P or H261L mutants). The total DNA amount was adjusted to 100 ng per well using an irrelevant non-transgene-expressing plasmid. 18-24 h after the second transfection 2/3 of the medium was removed from each well and substituted with either PBS pH 7.4 or acidic buffer (60 mM bis-tris pH 5.6, 150 mM NaCl). Cells were then incubated at 37°C for 1 h. After this, all medium was aspirated and replaced with 100 μL per well of fresh complete DMEM medium supplemented with 60 μM EnduRen substrate (Promega). Luminescence readings were performed at least 90 mins later using a Clariostar plate reader (BMG). Four replicate wells were assayed for the RVG-negative control condition. For WT, H261L and H270P constructs, four replicate wells (at pH 7.4) or eight replicate wells (at pH 5.6) were assayed for each of two transfections performed using independent DNA preparations.

Levels of expression of RABV-G were quantified by ELISA after the luciferase assay (data shown in Figure 4D lower graph). Cells were lysed with 20 μL per well of 0.5% polysorbate 20, 5 mM TrisHCl pH 7.4, 25 mM NaCl and 2.5% w/v sucrose. Lysates from each set of four or eight technical replicate wells were pooled and diluted to 400 μL in the same buffer. Maxisorp plates (ThermoFisher Scientific) were coated overnight with 100 ng per well of SO57-His Fab in PBS pH 7.5, washed with PBS pH 7.4 with 0.05% polysorbate 20 (PBS/T), and blocked for 1 h with CaseinBlocker (ThermoFisher Scientific). 50 uL of each sample was applied to each of three technical replicate wells for 1 h, washed with PBS/T. Bound RABV-G was detected using polyclonal serum from mice immunized with a licensed rabies vaccine (Rabipur, Valneva) as primary antibody, alkaline phosphatase conjugated goat anti-mouse IgG (Fc specific) as secondary antibody, p-nitrophenylphosphate substrate (both from Sigma), and a Clariostar plate reader (BMG). RABV-G content of each sample was quantified by interpolation against a standard curve of dilutions of purified RABV-G C-tag, prepared as described above.

Formation of syncytia was visualized with an Olympus SpinSR fluorescent microscope, using the same method as for luciferase detection with the exception of cells having been cultured in black 96-well plates (μ-Plate, Ibidi). Areas of 10x10 fields of view were acquired for each well using wide-field imaging at 20x magnification in bright-field and GFP channels. Images were analyzed with ImageJ software.

#### Competition ELISA

Maxisorp plates (ThermoFisher Scientific) were coated overnight with 50 ng per well of recombinant C-tagged RABV-G, diluted in PBS pH 7.4 with 1% n-octyl-β-d-glucoside, and blocked for 1 h with CaseinBlocker (ThermoFisher Scientific). One RABV-G antibody, in immunoglobulin G format (with the exception of RVC58 applied in Fab format), was applied at 20 μg/mL and incubated for 1 h. A second anti RABV-G antibody, in TwinStrep-tagged Fab format, was then applied at a gradient of concentrations and incubated for 1 h. After washing with PBS with 0.05% PBS/T, bound Fab was detected with horseradish-peroxidase-conjugated Streptactin and TMB substrate solution using a Clariostar plate reader (BMG).

#### Surface plasmon resonance

Surface plasmon resonance (SPR) measurements were made using a Biacore S200 instrument and software (Cytiva).

Neutral running buffer comprising 10 mM HEPES, 150 mM sodium chloride, 3 mM EDTA, and 0.05% polysorbate (HBS-EP+) was prepared and adjusted to pH 7.5. Acidic running buffer comprising 10 mM Bis-Tris, 150 mM sodium chloride, 3 mM EDTA, and 0.05% polysorbate (BBS-EP+) was prepared and adjusted to pH 5.6.

Single-cycle kinetics measurements of Fab – RABV-G interactions were performed using CM5 chips and amine coupling kit (both from Cytiva), at an analysis temperature of 4°C. Attempted capture of RABV-G using a C-terminal affinity tag was unsuccessful. To provide a surface which could be regenerated with fresh RABV-G for each cycle, we therefore used a sandwich configuration, whereby C-tagged RABV-G was first captured by another (non-competing) anti-RABV-G IgG antibody to produce an active flow cell, prior to application of the Fab of interest. For measurement of RVC20 kinetics, capture was on 17C7 at both pH 7.5 and 5.6. For measurement of 1112‑1 kinetics, capture was on RVC20 at pH 7.5, or on 17C7 at pH 5.6. For measurement of 17C7 kinetics, capture was on RVC20 at pH 7.5, or on 1112-1 at pH 5.6. Capture antibodies were immobilized at the maximum achievable densities (6000−12000 response unit, RU). An irrelevant IgG was immobilized at similar densities on a reference flow cell. At the start of each cycle, C-tagged wild-type RABV-G was applied to both active and reference cells, resulting in capture of approximately 100 RU on the active flow cells. Capture was presumed to be multi-valent and was stable, with dissociation of no more than 5.2% of bound RABV-G noted over 1650s (in control cycles in which buffer was used as analyte). For all three antibodies, recombinant Strep-tagged Fabs were used as analytes.

To collect an initial data set, at each of pH 5.6 and 7.5, one of the Fabs of interest was then injected at concentrations of 0.031, 0.125, 0.5, 2, 8, and 32 nM. No binding was observed for RVC20 at pH 5.6; in view of the presence of reduced binding for 1112-1 and 17C7 at pH 5.6, increased concentrations were subsequently used to improve data quality (1.15, 3.4, 10.3, 31, 93, 278 nM for 1112-1-TS Fab, and 0.27, 1.08, 4.34, 17.4, 69.5, 278 nM for 17C7-TS Fab). Each concentration of Fabs was injected for 240 s at a flow rate of 30 μL/min, followed by a final 1800 s dissociation phase. Data shown in Figure 4 is doubly-background-subtracted (i.e. after subtraction of responses on the irrelevant-IgG-coated reference flow cell and responses after injection of buffer). A 1:1 binding model was fitted using the Biacore S200 v2 Evaluation software. Extracted curves were plotted using Prism 9.0 (GraphPad software).

The ability of RABV-G to undergo pH-triggered conformational change after capture on a solid surface by chip-bound 17C7 and RVC58 (data shown in Figure 4) was tested with similar equipment, materials and chip preparation, with the exceptions of use of an analysis temperature of 21 ˚C and a T200 instrument and preparation of an additional chip for which RVC58 was immobilized on the active flow cell. The injection series was as indicated on the Figure 4 legend. RVC20 Fab was injected at fixed 0.33 μM concentration.

The ability of RABV-G to undergo pH-triggered conformational change after binding by 17C7 or RVC58 in solution (data shown in Figure S9E) was tested using a modified version of the above experimental design. RABV-G C-tag with A8-35 was prepared as above. It was then incubated for 10−15 mins either in pH 5.6 or pH 7.5 running buffer (as above), either with or without 15-fold molar excess of Fab (17C7 for panel E, RVC58 for panel F). Samples were then diluted to a final concentration of 1 μg/mL of RABV-G by the addition of >10 volumes of either acid or neutral running buffer. After a further 10 min incubation, the sample was applied for 60 s to an SPR chip. The difference in change in response units between an RVC20-coated flow cell and reference flow cell was calculated. Data is shown as a proportion of the level of binding seen at pH 7.5 in the presence of the antibody. Experiments were performed in singlicate.

### Quantification and statistical analysis

Figures 2, S5, and S9 present descriptive graphical comparisons of quantitative data. Data was analyzed and graphs produced using Prism 9.0 (GraphPad Software). The figure legends provide full details of the replication strategies and the measures of the location and dispersion of the data which are represented in the graphs. No statistical inference testing was performed. No assumptions were made which would require assessment of the appropriateness of a particular statistical approach.

## Data Availability

• A Cryo-EM density map with the corresponding atomic coordinates for the RABV-G – Fab 17C7 – Fab 1112-1 complex has been deposited in the Electron Microscopy Data Bank and Protein Data Bank and are publicly available as of the date of publication. Accession numbers are listed in the key resources table. • This paper does not report original code. • Any additional information required to reanalyze the data reported in this paper is available from the lead contact upon request.

## Supplemental information

Document S1. Figures S1–S9 and Tables S1–S3

Document S2. Article plus supplemental information
