# Supplementary material for: Structure of trimeric pre-fusion rabies virus glycoprotein in complex with two protective antibodies
Source: Cell Host Microbe. 2022 Sep 14;30(9):1219–1230.e7. doi: 10.1016/j.chom.2022.07.014 (PMC9605875; doi:10.1016/j.chom.2022.07.014)
Supplement: Document S1. Figures S1–S9 and Tables S1–S3 [file mmc1.pdf]

Cell Host & Microbe, Volume *30*

## Supplemental information

# Structure of trimeric pre-fusion rabies virus glycoprotein in complex with two protective antibodies

**Weng M. Ng, Sofiya Fedosyuk, Solomon English, Gilles Augusto, Adam Berg, Luke Thorley, Anna-Sophie Haselon, Rameswara R. Segireddy, Thomas A. Bowden, and Alexander D. Douglas**

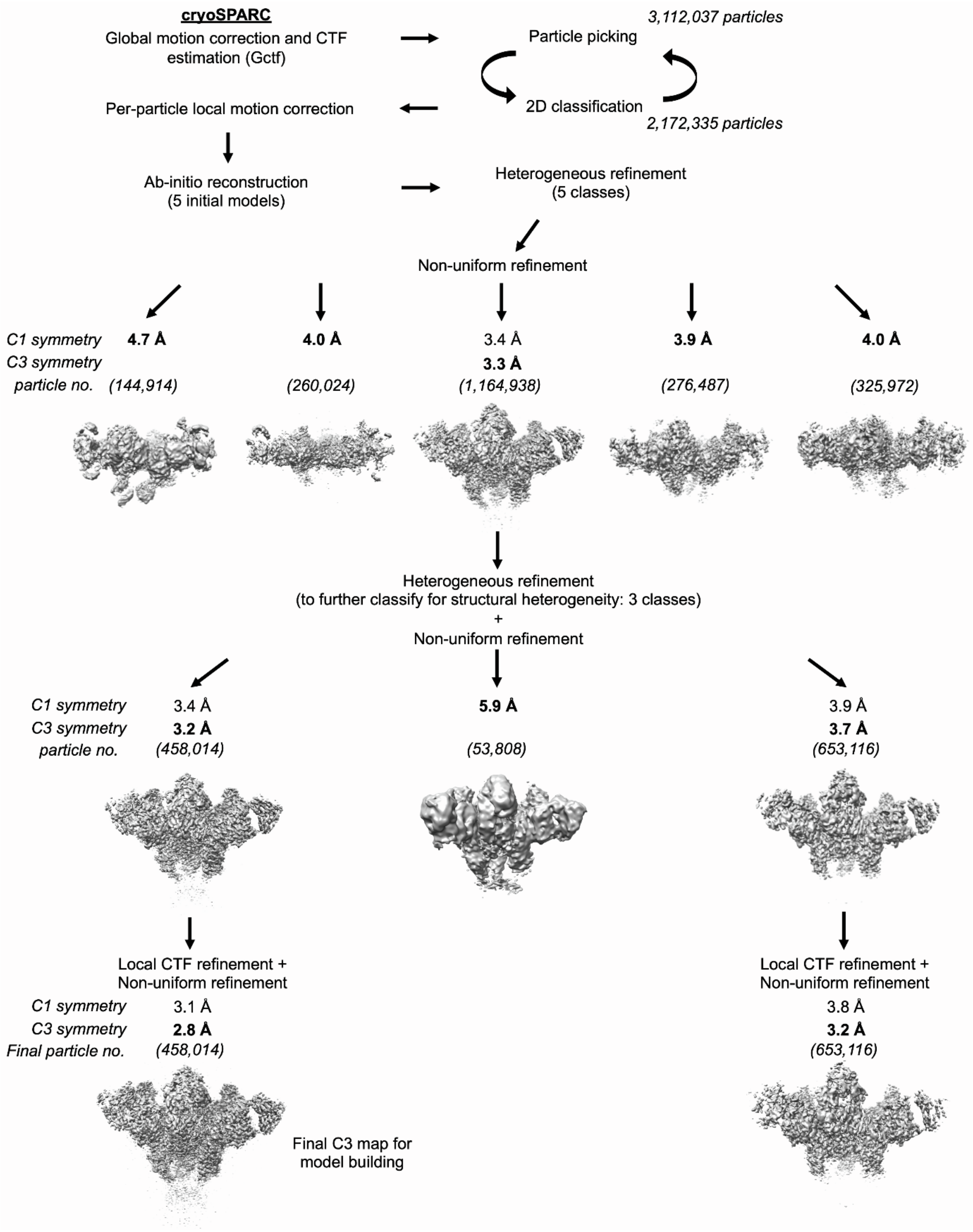


**Figure S1. Cryo-EM processing flowchart of RABV-G in complex with Fabs 17C7 and 1112-1, Related to Figure 1.** All processing steps were performed in cryoSPARC (Punjani et al., 2017). Cryo-EM maps (side view) reconstructed with non-uniform refinements are displayed as grey 3D volumes. Only C3 symmetry-applied maps are shown where both C1 and C3 symmetry-applied maps are available.

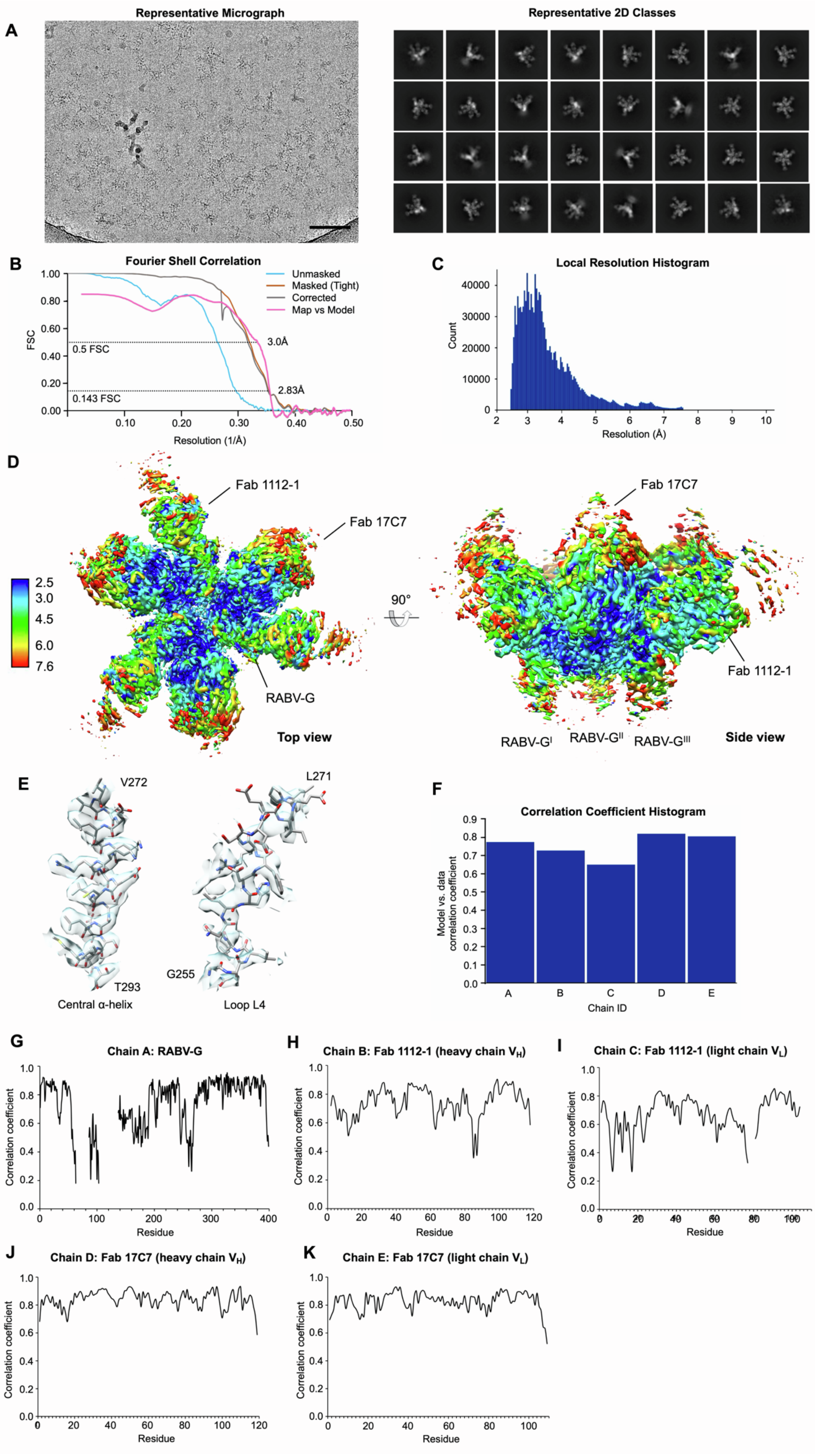

A
Representative Micrograph
Representative 2D Classes
B
Fourier Shell Correlation
Unmasked
Masked (Tight)
Corrected
Map vs Model
FSC
0.5 FSC
0.143 FSC
3.0Å
2.83Å
Resolution (1/Å)
C
Local Resolution Histogram
Count
Resolution (Å)
D
Fab 1112-1
Fab 17C7
RABV-G
Top view
90°
RABV-G^I
RABV-G^II
RABV-G^III
Side view
2.5
3.0
4.5
6.0
7.6
E
V272
T293
Central α-helix
L271
G255
Loop L4
F
Correlation Coefficient Histogram
Model vs. data correlation coefficient
Chain ID
G
Chain A: RABV-G
H
Chain B: Fab 1112-1 (heavy chain V_H)
I
Chain C: Fab 1112-1 (light chain V_L)
J
Chain D: Fab 17C7 (heavy chain V_H)
K
Chain E: Fab 17C7 (light chain V_L)
Correlation coefficient
Residue

**Figure S2. Resolution analysis of the cryo-EM structure of RABV-G in complex with Fabs 17C7 and 1112-1, Related to Figure 1.** (**A**) (*Left*) A representative cryo-EM micrograph of RABV-G – 17C7 – 1112-1 sample. The scale bar represents 10 nm. (*Right*) Representative 2D classes used in 3D reconstruction. (**B**) Fourier shell correlation (FSC) plots of reconstructions using gold-standard refinement in cryoSPARC (Punjani et al., 2017). Map resolutions were determined according to the 0.143 FSC cutoff. Curves are shown for unmasked (cyan), masked (brown) and corrected (grey) maps. FSC curve for the refined model versus the summed 2.83 Å map is shown in pink. (**C**) Local resolution histogram determined by cryoSPARC (Punjani et al., 2017). (**D**) Local resolution map estimated with cryoSPARC (Punjani et al., 2017). (**E**) Representative fit of atomic model into density. (**F**) Correlation coefficient per chain histogram determined by Phenix (Adams et al., 2002). (**G–K**) Correlation coefficient per residue graphs for (**G**) RABV-G, (**H**) Fab 1112-1 heavy chain $V_H$, (**I**) Fab 1112-1 light chain $V_L$, (**J**) Fab 17C7 heavy chain $V_H$, (**K**) Fab 17C7 light chain $V_L$.

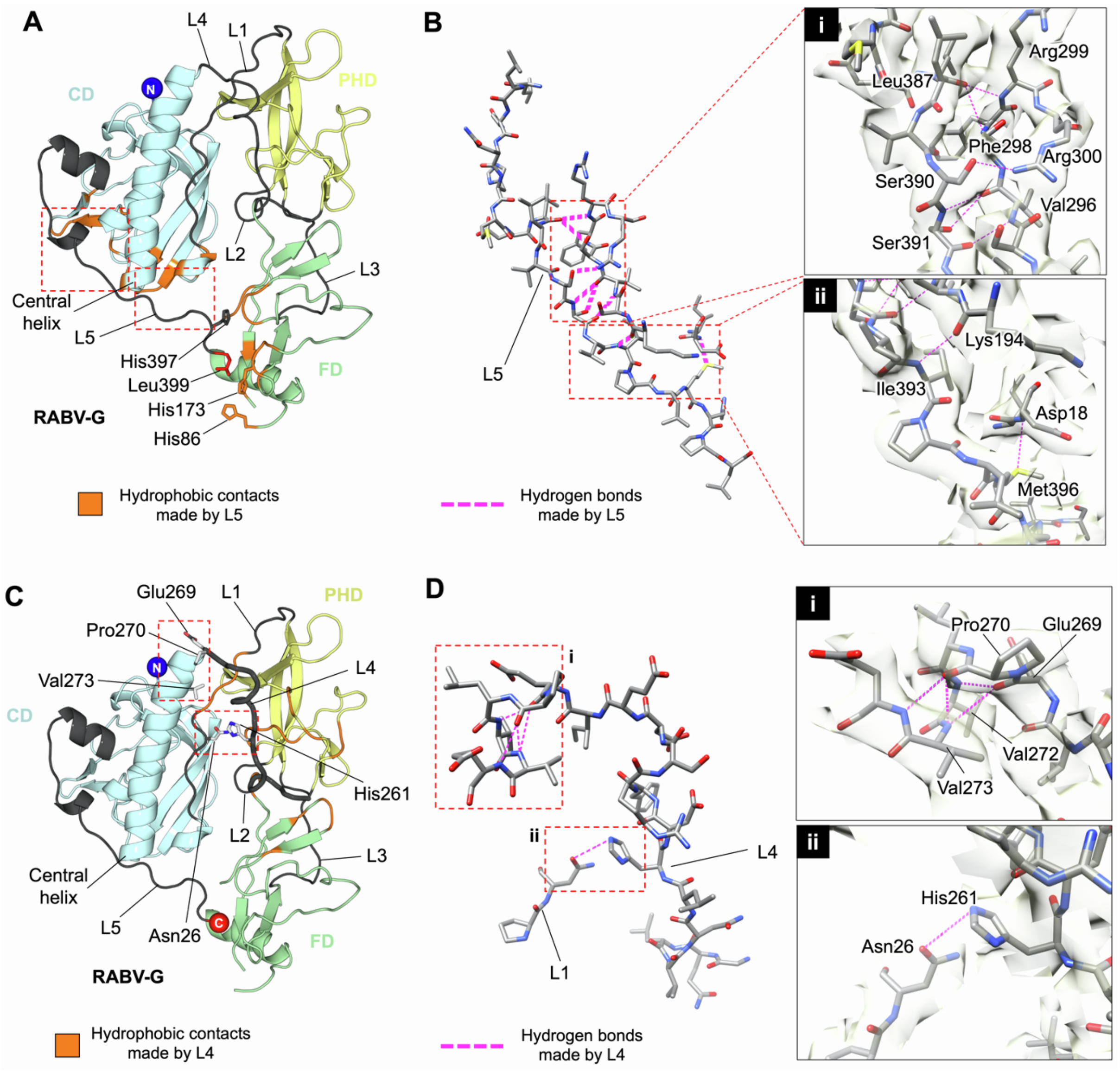


**Figure S3. Linkers L4 and L5 intra-protomeric contacts, Related to Figure 1.**

(**A**) Structure of a protomer of the RABV-G ectodomain crown is displayed in a cartoon representation, with PHD, CD, and FD colored yellow, cyan, and green, respectively. Inter-domain linkers L1–L5 are colored dark gray. The N and C-termini of the structure are shown as spheres and colored blue and red, respectively. Residues involved in intra-protomeric hydrophobic interactions with L5 are colored orange. Dashed line boxes indicate regions where L5 forms intra-protomeric hydrogen bonds. These regions are detailed in *panel B*.

(**B**) Intra-protomeric hydrogen bonds mediated by L5. Residues are shown in stick representation with carbon, nitrogen, oxygen, and sulfur atoms colored gray, blue, red, and yellow, respectively. Detailed interactions as indicated by the dashed line boxes are enlarged in sub-panels i–ii. Residues forming hydrogen bonds (pink dashed lines) are labeled. The cryo-EM map is shown with partial transparency. Interactions were determined using PDBePISA (Krissinel and Henrick, 2007).

(**C**) Structure of a protomer of the RABV-G ectodomain crown is shown as in *panel A*. L4 is labeled and shown with thicker width. Residues involved in intra-protomeric hydrophobic interactions with L4 are colored orange while residues involved in hydrogen bonding with L4 are shown as sticks.

(**D**) Detailed visualization of intra-protomeric hydrogen bonds formed by L4. Residues are displayed as sticks with carbon, nitrogen, and oxygen atoms colored gray, blue, and red, respectively. Detailed interactions as indicated by the dashed-line boxes are enlarged in sub-panels i–ii. Residues forming hydrogen bonds (pink dashed lines) are labeled. The cryo-EM map is shown with partial transparency. Interactions were determined using PDBePISA (Krissinel and Henrick, 2007).

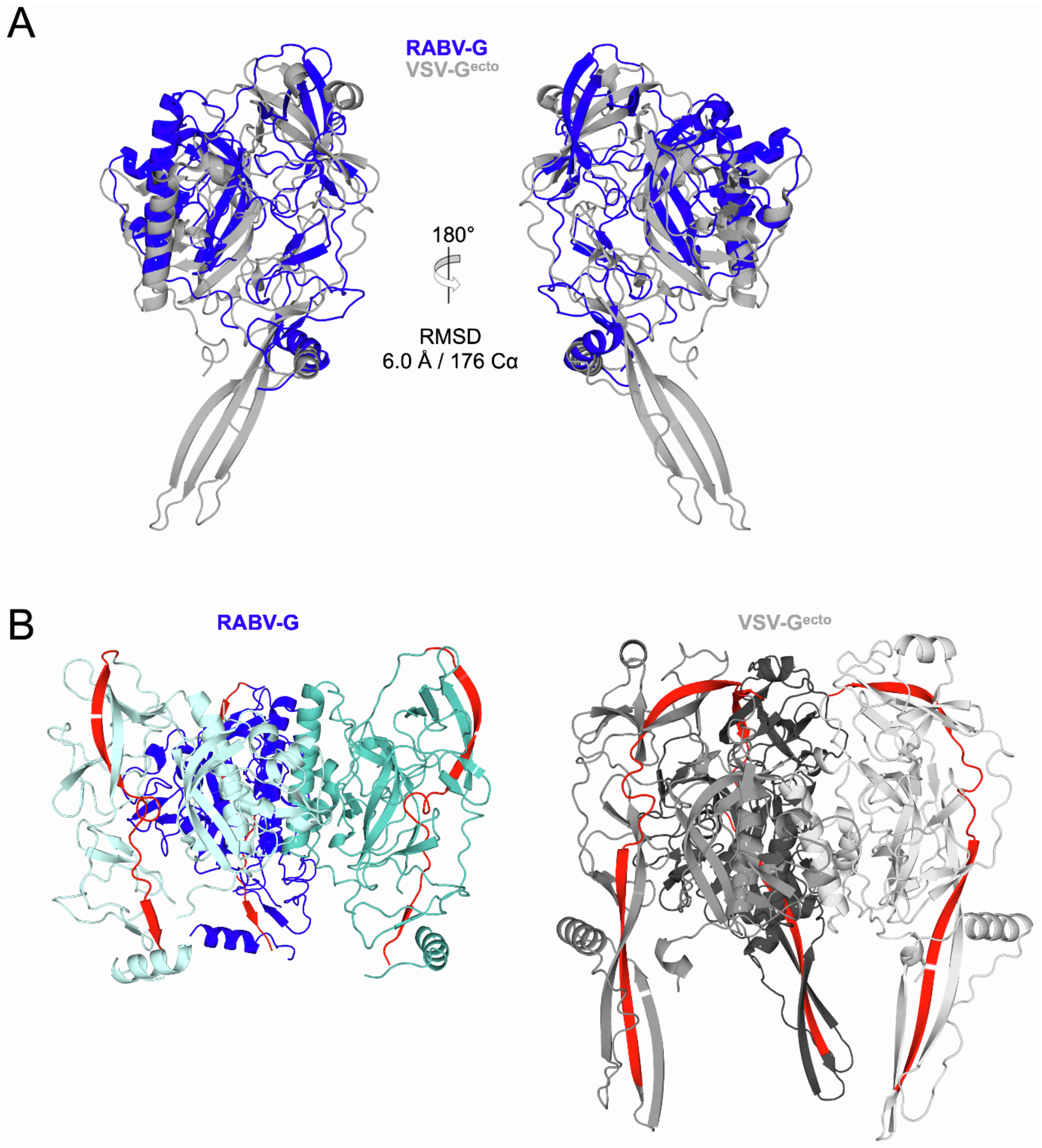


**Figure S4. Structural comparison of RABV-G and VSV-G, Related to Figure 3.**

(**A**) Structural superimposition of a single RABV-G protomer with VSV-G pre-fusion ectodomain protomer (PDB ID: 5I2S) (Roche et al., 2007). Structures are displayed in cartoon representation with RABV-G colored blue and VSV-G colored gray. Root-mean-square deviation (RMSD) of the alignment is indicated. Despite the relatively high RMSD of 6.0 Å, the overall conservation of the domain architecture can be seen.

(**B**) Differential angulations of RABV-G and VSV-G in the context of a trimer assembly. Trimeric RABV-G and VSV-G$^{ecto}$ are displayed as blue and gray cartoons, with different color shade for each protomer. Residues 36–63 in RABV-G and residues 36–69 in VSV-G$^{ecto}$ are colored red to show the angulations of the G molecules.

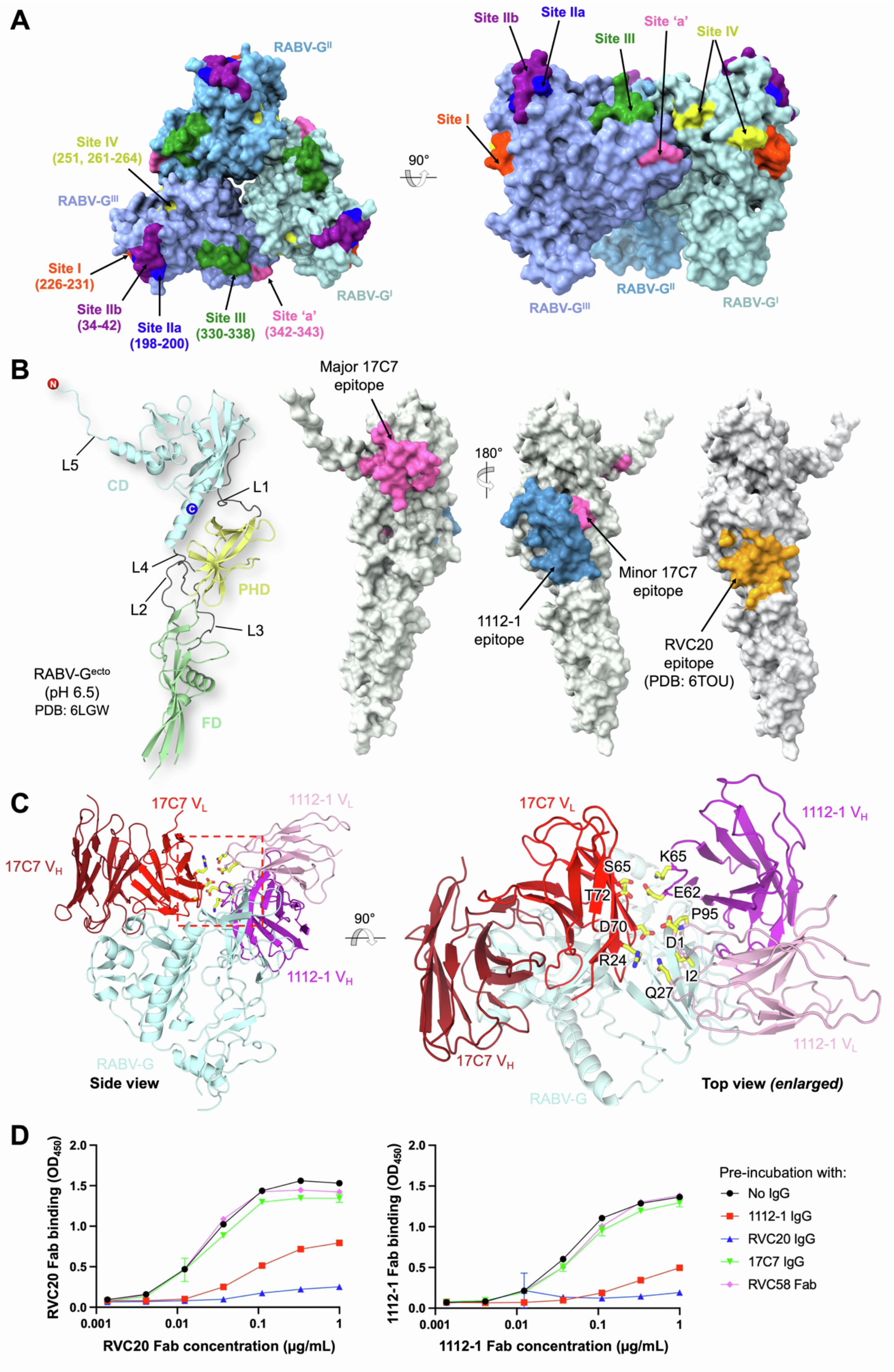

A
RABV-G$^{II}$
Site IV (251, 261-264)
RABV-G$^{III}$
Site I (226-231)
Site IIb (34-42)
Site IIa (198-200)
Site III (330-338)
Site 'a' (342-343)
RABV-G$^{I}$
90°
Site IIb
Site IIa
Site III
Site 'a'
Site IV
Site I
RABV-G$^{II}$
RABV-G$^{III}$
RABV-G$^{I}$
B
L5
CD
L1
L4
L2
PHD
L3
RABV-G$^{ecto}$ (pH 6.5) PDB: 6LGW
FD
Major 17C7 epitope
180°
Minor 17C7 epitope
1112-1 epitope
RVC20 epitope (PDB: 6TOU)
C
17C7 V$_L$
1112-1 V$_L$
17C7 V$_H$
1112-1 V$_H$
RABV-G
Side view
90°
S65
K65
T72
E62
D70
P95
D1
R24
I2
Q27
Top view (enlarged)
D
RVC20 Fab binding (OD$_{450}$)
RVC20 Fab concentration (μg/mL)
1112-1 Fab binding (OD$_{450}$)
1112-1 Fab concentration (μg/mL)
0.0
0.5
1.0
1.5
2.0
0.001
0.01
0.1
1
Pre-incubation with:
No IgG
1112-1 IgG
RVC20 IgG
17C7 IgG
RVC58 Fab

**Figure S5. RABV-G antigenic sites, Related to Figure 4.**

(**A**) Characterized antigenic sites I–IV and 'a' (Kuzmina et al., 2013) are mapped onto the trimeric RABV-G structure (surface representation, with each protomer in different shades of blue). Residue numbers for each site are indicated in parentheses.

(**B**) (*Left*) Crystal structure of RABV-G$^{ecto}$ obtained at pH 6.5 and hence believed to represent post-fusion conformation (PDB ID: 6LGW) (Yang et al., 2020) displayed as cartoon with CD, PHD, FD, and L1–L5 colored and labeled accordingly. (*Right*) Footprints of 17C7 and 1112-1 epitopes determined in this study are mapped onto the RABV-G$^{ecto}$ (pH 6.5) crystal structure (white, surface representation). 17C7 footprint, pink; 1112-1 footprint, blue. Residues previously shown to form contacts with RVC20 are shown in orange – this epitope is not believed to be accessible in post-fusion conformation (Hellert et al., 2020).

(**C**) Contacts observed between Fab 1112-1 and Fab 17C7 in the structure. A RABV-G monomer (light blue) is shown with the variable regions of Fab 1112-1 (purple/pink) and 17C7 (dark/bright red). Top view for the complex is enlarged to show the contacts between Fab 1112-1 $V_H/V_L$ and Fab 17C7 $V_L$, with the interfacing residues shown as sticks and labeled. Contacts between the two Fabs constitute approximately 130 Å$^2$ of interaction interface, as determined by PDBePISA (Krissinel and Henrick, 2007).

**(D)** Competitive binding between 17C7, 1112-1, RVC20, and RVC58 to RABV-G, as determined by ELISA. Results indicated that antibody pairs 17C7/RVC20, RVC58/RVC20, 17C7/1112-1, RVC58/1112-1 are compatible for RABV-G co-binding, but not RVC20/1112-1. Plates coated with recombinant RABV-G–C-tag were pre-incubated with 20 µg/mL of the indicated antibodies, before application of TwinStrep-tagged Fab 1112-1 or Fab RVC20 and detection with horseradish-peroxidase-conjugated Streptactin. Points and error bars represent median and range of triplicate wells (technical replicates).

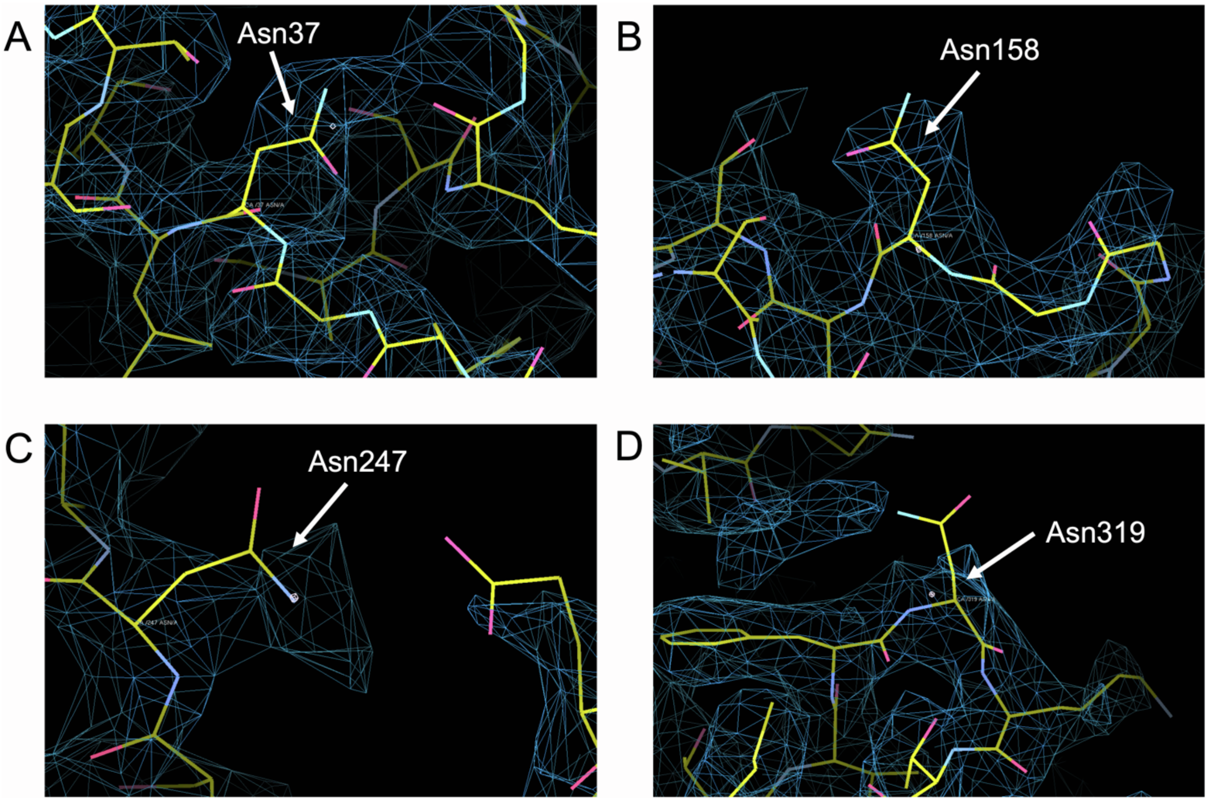


**Figure S6. Densities corresponding to asparagine (N)-linked glycosylation sequons within RABV-G, Related to Figure 4.**

(**A**)–(**D**) Atomic model of RABV-G displaying N-linked glycosylation sequons (NXT/S, X ≠ P). Amino acid residues are shown in stick representation, with carbon, nitrogen, oxygen, and sulfur atoms colored yellow, blue, pink, and dark yellow, respectively. Asn37, Asn158, Asn247, and Asn319 are labeled. Map densities are shown as blue mesh, rendered in Coot (Emsley and Cowtan, 2004). No ordered densities corresponding to glycans were observed in our map, but we are unable to distinguish between disordered glycans and unoccupied sequons.

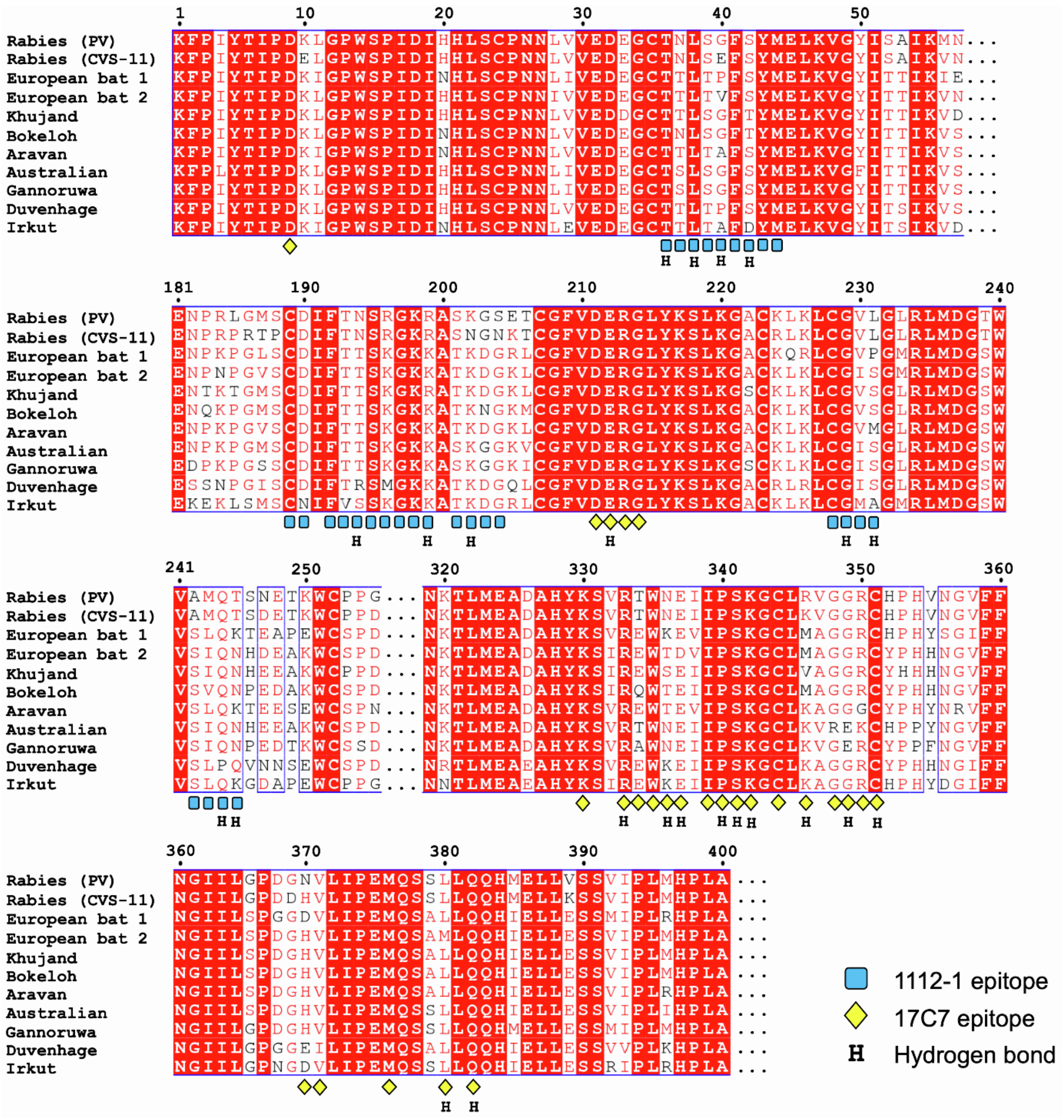

**Figure S7. Conservation and variation of 17C7 and 1112-1 contact residues across lyssaviruses, Related to Figure 4.** Glycoprotein amino acid sequences of PV strain rabies virus(Kim et al., 2016), CVS-11 rabies virus (ADJ29911.1), European bat lyssavirus 1 (YP_001285391.1), European bat lyssavirus 2 (YP_001285396.1), Khujand virus (YP_009094330.1), Bokeloh bat lyssavirus (YP_009091812.1), Aravan lyssavirus (YP_007641395.1), Australian bat lyssavirus (QIN55368.1), Gannoruwa lyssavirus (YP_009325517.1), Duvenhage lyssavirus (YP_007641405.1), and Irkut lyssavirus (AFP74571.1) were determined using MultAlin(Corpet, 1988) and plotted with ESPript(Gouet et al., 2003). Identical residues are shaded in red. Residues interacting with Fabs 1112-1 and 17C7 in our structure were identified using PDBePISA (Krissinel and Henrick, 2007) and are annotated beneath the alignment as indicated. Residues forming hydrogen bonds with the Fab fragments are denoted 'H'.

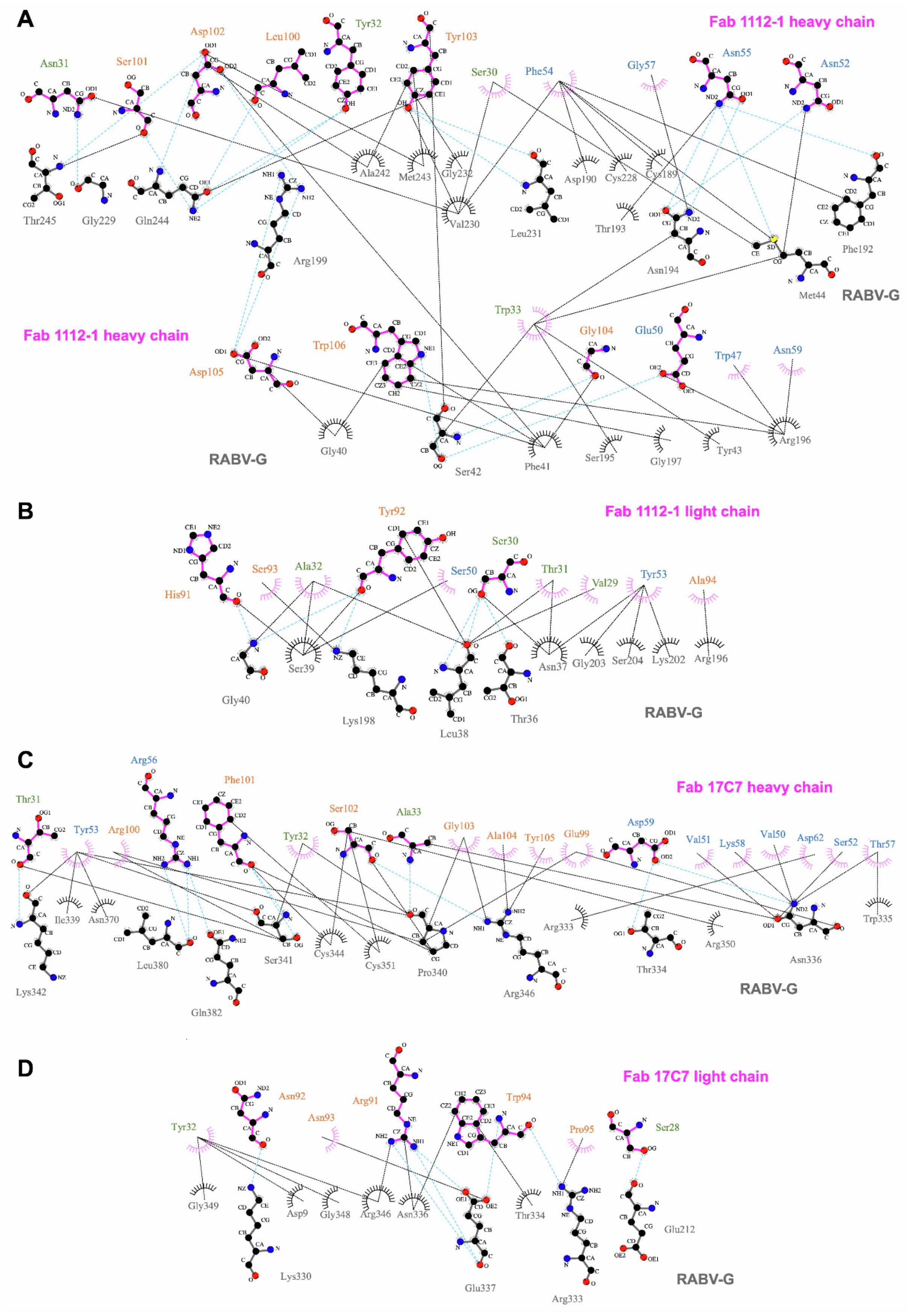

A
Fab 1112-1 heavy chain
Asn31
Ser101
Asp102
Leu100
Tyr32
Tyr103
Ser30
Phe54
Gly57
Asn55
Asn52
Thr245
Gly229
Gln244
Arg199
Ala242
Met243
Gly232
Val230
Leu231
Asp190
Cys228
Cys189
Thr193
Asn194
Phe192
Met44
RABV-G
Fab 1112-1 heavy chain
Asp105
Trp106
Trp33
Gly104
Glu50
Trp47
Asn59
RABV-G
Gly40
Ser42
Phe41
Ser195
Gly197
Tyr43
Arg196
B
Fab 1112-1 light chain
His91
Ser93
Ala32
Tyr92
Ser50
Ser30
Thr31
Val29
Tyr53
Ala94
Gly40
Ser39
Lys198
Leu38
Thr36
Asn37
Gly203
Ser204
Lys202
Arg196
RABV-G
C
Fab 17C7 heavy chain
Thr31
Arg56
Phe101
Tyr53
Arg100
Tyr32
Ser102
Ala33
Gly103
Ala104
Tyr105
Glu99
Asp59
Val51
Lys58
Val50
Asp62
Ser52
Thr57
Ile339
Asn370
Lys342
Leu380
Gln382
Ser341
Cys344
Cys351
Pro340
Arg346
Arg333
Thr334
Arg350
Asn336
Trp335
RABV-G
D
Fab 17C7 light chain
Tyr32
Asn92
Asn93
Arg91
Trp94
Pro95
Ser28
Gly349
Lys330
Asp9
Gly348
Arg346
Asn336
Glu337
Thr334
Arg333
Glu212
RABV-G

**Figure S8. Schematic diagram of contacts formed at the RABV-G – Fab 1112-1 and RABV-G – Fab 17C7 interfaces, Related to Figure 4.** (**A**) RABV-G–Fab 1112-1 heavy chain, (**B**) RABV-G–1112-1 light chain, (**C**) RABV-G–17C7 heavy chain, and (**D**) RABV-G–17C7 light chain interactions. RABV-G residues are colored gray and Fab residues are colored magenta. Labels for Fab CDR residues are colored green, blue, and orange to represent CDR H1/L1, H2/L2, and H3/L3, respectively. Atoms corresponding to carbon, nitrogen, and oxygen are shown as black, blue, and red balls, respectively. Residues involved in hydrogen bonding are shown as sticks; residues involved in hydrophobic interactions are shown as spoked arcs. Hydrogen bonds (hydrogen-acceptor and donor-acceptor distance range of 2.70-3.35 Å) and hydrophobic interactions (distance range of 2.9-3.9 Å) are shown as cyan and black dotted lines, respectively. Plots were generated with LigPlot+ (Laskowski and Swindells, 2011).

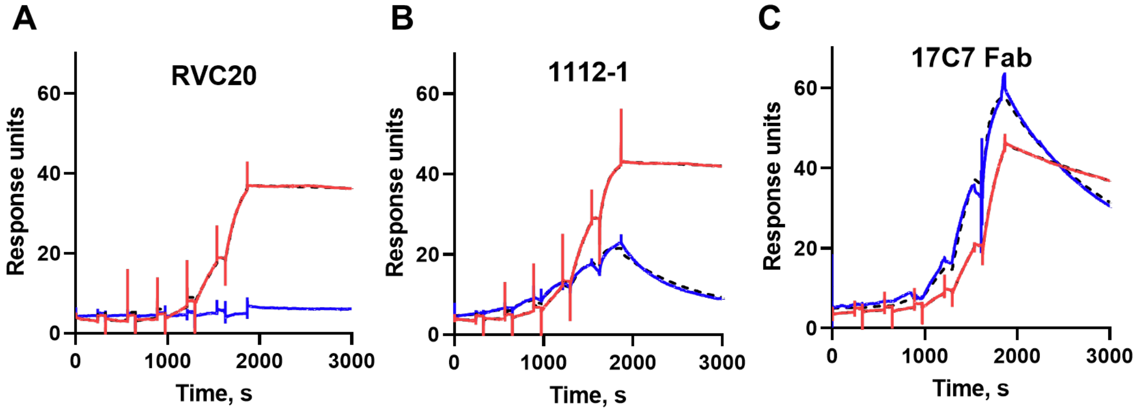


D

| Fab | Buffer pH | ka (1/Ms) | kd (1/s) | $K_D$ (nM) |
|---|---|---|---|---|
| RVC20 | pH 7.5 | $2.0x10^5$ | $2.0x10^{-5}$ | 0.01 |
| | pH 5.6 | none | none | none |
| 1112-1 | pH 7.5 | $3.8x10^5$ | $2.4x10^{-5}$ | 0.06 |
| | pH 5.6 | $8.0x10^4$ | $1.2x10^{-3}$ | 15 |
| 17C7 | pH 7.5 | $1.4x10^5$ | $2.2x10^{-4}$ | 1.6 |
| | pH 5.6 | $7.8x10^4$ | $9.7x10^{-4}$ | 13 |

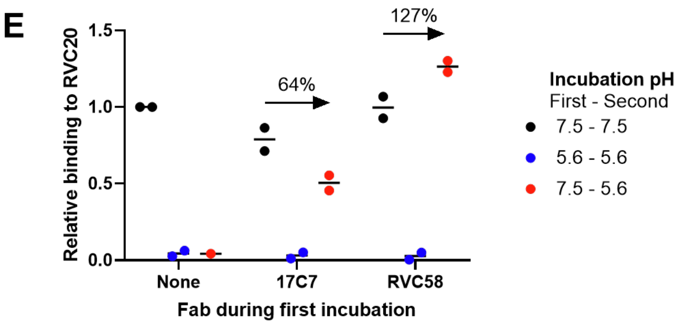


**Figure S9. RABV-G interactions with Fabs and antibodies under acidic and neutral pH conditions, Related to Figure 4.**

**(A–C)** SPR analysis of the kinetics of RVC20, 1112-1 and 17C7 interactions with RABV-G. In each case data observed at pH 7.4 and pH 5.6 are represented by red and blue lines respectively. Calculated kinetic and affinity values are shown in **(D).** Fab concentration ranges used differed between cycles, as described in methods.

(**E**) Antibodies 17C7 and RVC58 inhibit pH-triggered conformational change after binding RABV-G in solution. RABV-G was first incubated at pH 7.5 or 5.6, either without antibody, with 17C7 Fab, or with RVC58 Fab. The sample then underwent a second incubation after dilution with buffer at either pH 7.5 or 5.6 (including a condition in which samples underwent the first incubation at pH 7.5 and second incubation at pH 5.6). Binding to RVC20 was then measured by SPR.

Points indicate data from two independent experiments (with the exception of the no antibody : pH 7.6 – 5.6 condition, which was performed in singlicate), with lines indicating the median. RABV-G : RVC20 binding, signifying pre-fusion conformation, is shown relative to that observed in each experiment at pH 7.5 in the absence of any antibody. Colors of points indicate incubation pH, as per the legend.

64% and 127% indicate the proportion of RVC20 binding preserved after lowering of pH to 5.6 following initial incubation at pH 7.5 with 17C7 or RVC58 respectively (with 100% denoting binding of samples incubated at pH 7.5 throughout, in the presence of the same antibody). The same pH change reduced binding to 4% in absence of any antibody.

**Table S1. Monoclonal antibodies used in study, Related to Figures 1, 2 and 4.**

Antibodies are tabulated in the order in which they are mentioned in the text.

Antigenic sites, and results of competition ELISA to assess co-binding of antibody pairs, are shown in Figure S5.

| **Antibody** | **Antigenic site** | **Other characteristics and role in study** | **Reference(s)** |
|---|---|---|---|
| 17C7 (also known as RAB1) | III | • Protective against RABV-G in pre-clinical challenge.<br>• Licensed in India for post-exposure prophylaxis as Rabishield.<br>• Component of complex used for cryo-EM (Figures 1, 3, 4)<br>• Confirmation of conformational accuracy of RABV-G mutants (Figure 2)<br>• Mechanism explored (Figure 4) | (Sloan et al., 2007) |
| 1112-1 | II | • Protective against RABV-G in pre-clinical challenge.<br>• Has been considered a candidate for inclusion in post-exposure prophylactic antibody cocktail.<br>• Component of complex used for cryo-EM (Figures 1, 3, 4)<br>• Confirmation of conformational accuracy of RABV-G mutants (Figure 2) | (Dietzschold et al., 1992; Muller et al., 2009) |
| RVC20 | I | • Previously shown to be specific for pre-fusion conformation of RABV-G, and suggested to lock RABV-G in this conformation.<br>• Used here as probe for pre-fusion conformation of RABV-G (Figure 2, Figure 4D) | (De Benedictis et al., 2016; Hellert et al., 2020) |
| RVC58 | III | • Demonstration of blockade of RABV-G conformational transition by a second site III-binding antibody, in addition to 17C7 (Figure 4D). | (De Benedictis et al., 2016) |

**Table S2. Cryo-EM data collection, processing, and model refinement statistics, Related to Figure 1.**

| | |
|---|---|
| **Data collection and processing** | |
| Microscope | Titan Krios |
| Detector | Gatan K3 |
| Voltage (kV) | 300 |
| Recording mode | Super resolution |
| Electron dose ($e^-/Å^2$) | 44.4 |
| Defocus range (µm) | –0.8 to –2.6 |
| Frames | 45 |
| Magnification | 81,000 |
| Final map pixel size (Å/px) | 1.06 |
| Symmetry imposed | C3 |
| No. of movies | 12,884 |
| No. of final particles images | 458,014 |
| Map resolution at 0.143 FSC threshold (Å) | 2.8 |
| Map sharpening B-factor ($Å^2$) | −108 |
| **Model refinement**[a] | |
| FSC model vs. map at 0.5 threshold (Å) | 3.0 |
| CC model vs. map (masked) | 0.80 |
| *Model composition* | |
| Non-hydrogen atoms | 6,134 |
| Protein residues | 790 |
| Non-protein residues | 0 |
| *R.m.s. deviations* | |
| Bond lengths (Å) | 0.003 |
| Bond angles (°) | 0.546 |
| *Validation* | |
| Molprobity score | 1.80 |
| Clashscore | 7.93 |
| Poor rotamers (%) | 0.00 |
| Cβ outliers (%) | 0.00 |
| Cis-proline (%) | 7.30 |
| Cis-general (%) | 0.00 |
| Twisted proline (%) | 0.00 |
| Twisted general (%) | 0.00 |
| *Ramachandran plot* | |
| Favored (%) | 94.70 |
| Allowed (%) | 5.30 |
| Outliers (%) | 0.00 |

[a]Refinement statistics correspond to a RABV-G–17C7–1112-1 monomer. Trimeric assembly was generated with experimental map-derived C3 symmetry.

**Table S3. Cell-surface expression levels of mutant RABV-G constructs, Related to Figure 2.** Table reports cell-surface expression level of each tested RABV-G construct. Median fluorescence intensity after staining with labelled RVC20 was measured, and is reported as a proportion of that observed with the appropriate wildtype (WT) comparator (i.e. untagged WT for untagged constructs, GFP-tagged WT for GFP-tagged constructs). Table reports median and range of four technical replicates across two experiments (a transfection with each of two independent DNA preparations on each of two days).

| Construct details | | | | | Cell-surface expression (proportion of WT) | | |
|---|---|---|---|---|---|---|---|
| **Plasmid reference number** | **Residue number** | **Changed from** | **Changed to** | **Untagged or GFP fusion?** | **Median** | **Upper limit of range** | **Lower limit of range** |
| ADP502 | 20 | H | A | Untagged | 0.98 | 1.27 | 0.84 |
| ADP514 | 20 | H | L | GFP | 0.56 | 0.76 | 0.33 |
| ADP503 | 21 | H | A | GFP | 1.24 | 1.69 | 0.81 |
| ADP515 | 21 | H | L | GFP | 0.20 | 0.23 | 0.18 |
| ADP504 | 86 | H | A | Untagged | 0.08 | 0.15 | 0.06 |
| ADP516 | 86 | H | L | GFP | 0.17 | 0.34 | 0.13 |
| ADP505 | 113 | H | A | GFP | 0.84 | 1.53 | 0.20 |
| ADP517 | 113 | H | L | GFP | 0.72 | 2.24 | 0.35 |
| ADP506 | 150 | H | A | Untagged | 0.88 | 0.99 | 0.70 |
| ADP518 | 150 | H | L | GFP | 0.70 | 0.81 | 0.44 |
| ADP507 | 173 | H | A | GFP | 0.05 | 0.06 | 0.04 |
| ADP519 | 173 | H | L | Untagged | 0.02 | 0.02 | 0.02 |
| ADP508 | 261 | H | A | Untagged | 0.91 | 1.13 | 0.83 |
| ADP596 | 261 | H | L | Untagged | 1.01 | 1.07 | 0.75 |
| ADP520 | 261 | H | L | GFP | 0.80 | 1.04 | 0.72 |
| ADP600 | 266 | D | P | Untagged | 1.23 | 1.42 | 1.03 |
| ADP599 | 268 | I | P | Untagged | 0.87 | 1.09 | 0.74 |
| ADP484 | 269 | E | P | GFP | 0.04 | 0.05 | 0.03 |
| ADP593 | 270 | H | P | Untagged | 1.04 | 1.40 | 0.80 |
| ADP485 | 270 | H | P | GFP | 1.38 | 2.45 | 1.07 |
| ADP594 | 271 | L | P | Untagged | 1.10 | 2.02 | 0.90 |
| ADP486 | 271 | L | P | GFP | 1.05 | 1.69 | 0.82 |
| ADP595 | 272 | V | P | Untagged | 1.12 | 1.29 | 0.82 |
| ADP487 | 272 | V | P | GFP | 1.16 | 1.50 | 0.84 |
| ADP488 | 273 | V | P | GFP | 1.78 | 2.10 | 1.12 |
| ADP509 | 303 | H | A | GFP | 0.59 | 0.70 | 0.46 |
| ADP521 | 303 | H | L | Untagged | 0.93 | 1.22 | 0.79 |
| ADP510 | 328 | H | A | Untagged | 0.10 | 0.13 | 0.05 |
| ADP614 | 384 | H | K | Untagged | 0.91 | 1.22 | 0.62 |
| ADP601 | 384 | H | P | Untagged | 1.08 | 1.27 | 0.81 |
| ADP511 | 397 | H | A | Untagged | 0.09 | 0.17 | 0.06 |
| ADP523 | 397 | H | L | GFP | 0.05 | 0.06 | 0.04 |
| ADP512 | 419 | H | A | Untagged | 0.02 | 0.02 | 0.02 |
| ADP524 | 419 | H | L | Untagged | 1.03 | 1.31 | 0.67 |
| ADP513 | 424 | H | A | GFP | 0.44 | 0.90 | 0.14 |
| ADP525 | 424 | H | L | Untagged | 1.13 | 1.28 | 0.24 |
| ADP079 | WT | | | Untagged | 1.00 | 1.12 | 0.88 |
| ADP427 | WT | | | GFP | 1.00 | 1.17 | 0.83 |
